# Dynamic changes in clinicopathological variables and gene expression in dogs with immune-mediated hemolytic anemia

**DOI:** 10.1371/journal.pone.0359710

**Published:** 2026-10-28

**Authors:** James W. Swann, Sivert Nerhagen, Balazs Szladovits, Gina Parkes, Anna Riddle, Samantha Mason, Brian Catchpole, Yu-Mei Chang, Barbara Glanemann

**Affiliations:** 1 Department of Clinical Science and Services, Royal Veterinary College, Hatfield, United Kingdom; 2 Columbia Stem Cell Initiative, Columbia University, New York, New York, United States of America; 3 Department of Pathobiology and Population Sciences, Royal Veterinary College, Hatfield, United Kingdom; 4 Clinical Investigation Centre, Royal Veterinary College, Hatfield, United Kingdom; 5 Department of Comparative Biomedical Sciences, Royal Veterinary College, London, United Kingdom; Abbvie Biotherapeutics Inc, UNITED STATES OF AMERICA

## Abstract

**Background:**

Immune-mediated hemolytic anemia (IMHA) is a common autoimmune disease of dogs that is difficult to treat effectively. Little is known about how clinicopathological and immune variables change with time in dogs with IMHA, and whether these changes could guide therapeutic interventions.

**Aims:**

To investigate dynamic changes in clinicopathological variables as well as immune and glucocorticoid-regulated gene expression in dogs with IMHA, and to determine whether these changes are associated with recovery from anemia and severity of treatment-related adverse effects.

**Animals:**

29 client-owned dogs with non-associative IMHA.

**Methods:**

Dogs with IMHA underwent clinical monitoring, with collection of residual blood samples at serial time points. Expression of immune and glucocorticoid-regulated genes was quantified using real time quantitative polymerase chain reactions, with serum cytokine concentrations measured using multiplex arrays.

**Results:**

Several clinical and gene expression variables changed with time in dogs with IMHA undergoing treatment, and we identified 6 major clusters representing different patterns of temporal change. No candidate dynamic variables were associated with more rapid recovery from anemia, but early increases in serum alkaline phosphatase (ALP) activity were associated with severity of later polydipsia.

**Conclusions:**

Immune and clinicopathological variables are highly changeable after onset of treatment in dogs with IMHA, with early increases in serum ALP activity being associated with later severity of polydipsia. This parameter is therefore a key candidate for further investigations of individualization of immunosuppressive therapy for dogs with IMHA.

## Introduction

Immune-mediated hemolytic anemia (IMHA) is among the most common autoimmune diseases of dogs [1], with most cases considered non-associative because no underlying cause is found upon clinical evaluation [2]. Loss of immune tolerance results in autoreactive lymphocyte responses and production of autoantibodies directed against red blood cell (RBC) antigens, leading to hemolysis [3]. Despite advances in supportive care, treatment of dogs with IMHA is challenging and often necessitates prolonged therapy with broad-spectrum immunosuppressive drugs, especially glucocorticoids such as prednisone/prednisolone [4]. Although often effective in controlling the autoimmune response, treatment with glucocorticoids at high dosages and for prolonged periods frequently causes marked adverse effects (AE) in dogs, including polydipsia, polyuria, excessive panting, weight gain, and loss of muscle mass [5–7].

Typically, glucocorticoids are administered at fixed dosages to dogs with IMHA after diagnosis, followed by empirical dose reductions over several months [8]. A previous pilot study showed that simple modifications to the dosing regimen, such as fractionation of the daily glucocorticoid dose, can produce more rapid improvements in polydipsia without compromising control of the autoimmune response in dogs with IMHA [9]. However, it remains challenging to predict which dogs will experience severe glucocorticoid-related AEs, thus limiting the ability of clinicians to individualize glucocorticoid regimens to different dogs with IMHA. Dogs with IMHA are often subjected to careful monitoring through clinical examination and serial clinicopathological testing, leading to the notion that early dynamic changes in some variables might predict expeditious recovery from anemia or severity of AEs later in the treatment timeline. Additionally, glucocorticoids suppress production of T cell-derived inflammatory cytokines, such as interleukin (IL)-2, IL-17, and interferon (IFN)-γ, and previous studies have shown that expression of the genes encoding some of these molecules in stimulated peripheral blood can be used to monitor the effectiveness of immunosuppression [10,11]. Moreover, studies in human cells have identified promoters bound by the glucocorticoid receptor and upregulated upon dexamethasone exposure, including *Zfp36*, *Dusp1*, *Nfkbia*, and *Cyb5a* [12,13], which therefore also represent promising candidates for evaluating glucocorticoid responses. Based on these data, we hypothesized that evaluation of dynamic measures of early glucocorticoid response using clinicopathological monitoring or expression of cytokine or glucocorticoid-regulated genes could represent new clinical tools to assist veterinarians in selecting and adjusting appropriate immunosuppressive dose regimens for individual dogs.

To address this outstanding clinical need, we conducted a prospective, observational study of dogs with non-associative IMHA, in which the major aims were (1) to describe dynamic changes in clinicopathological, immune, and gene expression variables after onset of treatment, and (2) to evaluate whether dynamic changes were associated with recovery from anemia or severity of treatment-induced AEs.

## Results

### Different clinical features show distinct patterns of temporal change in dogs with IMHA

To evaluate dynamic changes in clinical features as possible predictors of recovery from anemia or severity of treatment-related AEs in dogs with IMHA, we conducted a prospective, observational study, in which we evaluated dogs at multiple times after diagnosis, as shown in Fig 1. A total of 80 dogs were considered for inclusion in the study, of which 4 were excluded because they had non-regenerative immune-mediated anemia or pure red cell aplasia, 2 because they were considered to have IMHA caused by underlying disease (n = 1 cholecystitis and 1 with right auricular mass), and 1 that had a blood transfusion prior to presentation at the study institution. Of the 73 dogs enrolled in the study, 44 were excluded from analysis because they had incomplete residual samples (n = 16), died or were euthanized before the first follow-up visit owing to clinical deterioration (13), were lost to follow-up or had poor compliance to treatment directions before the first follow-up visit (8), required additional interventions while hospitalized (5), developed an unexpected complication of septic peritonitis in the first week after starting treatment (1), or was not anemic at presentation despite having features of immune-mediated RBC destruction (1), leaving 29 dogs for analysis (S1 Fig). Included dogs displayed typical clinical features of IMHA, with a median packed cell volume (PCV) of 13% (inter-quartile range [IQR]: 11–16, reference interval [RI]: 35–55) and median serum total bilirubin of 1.22 mg/dL (IQR: 0.62-1.94, RI: 0.01-0.25). Enrolled dogs had a median age of 100 months (IQR: 60–115), and there was a preponderance of female dogs (female intact n = 1, 3.4%; female neutered n = 18, 62.1%; male intact n = 1, 3.4%; and male neutered n = 9, 31.0%). Cross breeds were the most frequent breed (n = 7, 24.1%), followed by Cocker spaniels, Springer spaniels, West Highland white terriers, Labrador retrievers, Bichon frise, and Maltese terriers (each n = 2, 6.9%). A number of other breeds were included (n = 10 dogs), and complete summaries of demographic and presenting clinical features are shown in S1*–*S2 Tables.

**Fig 1:**
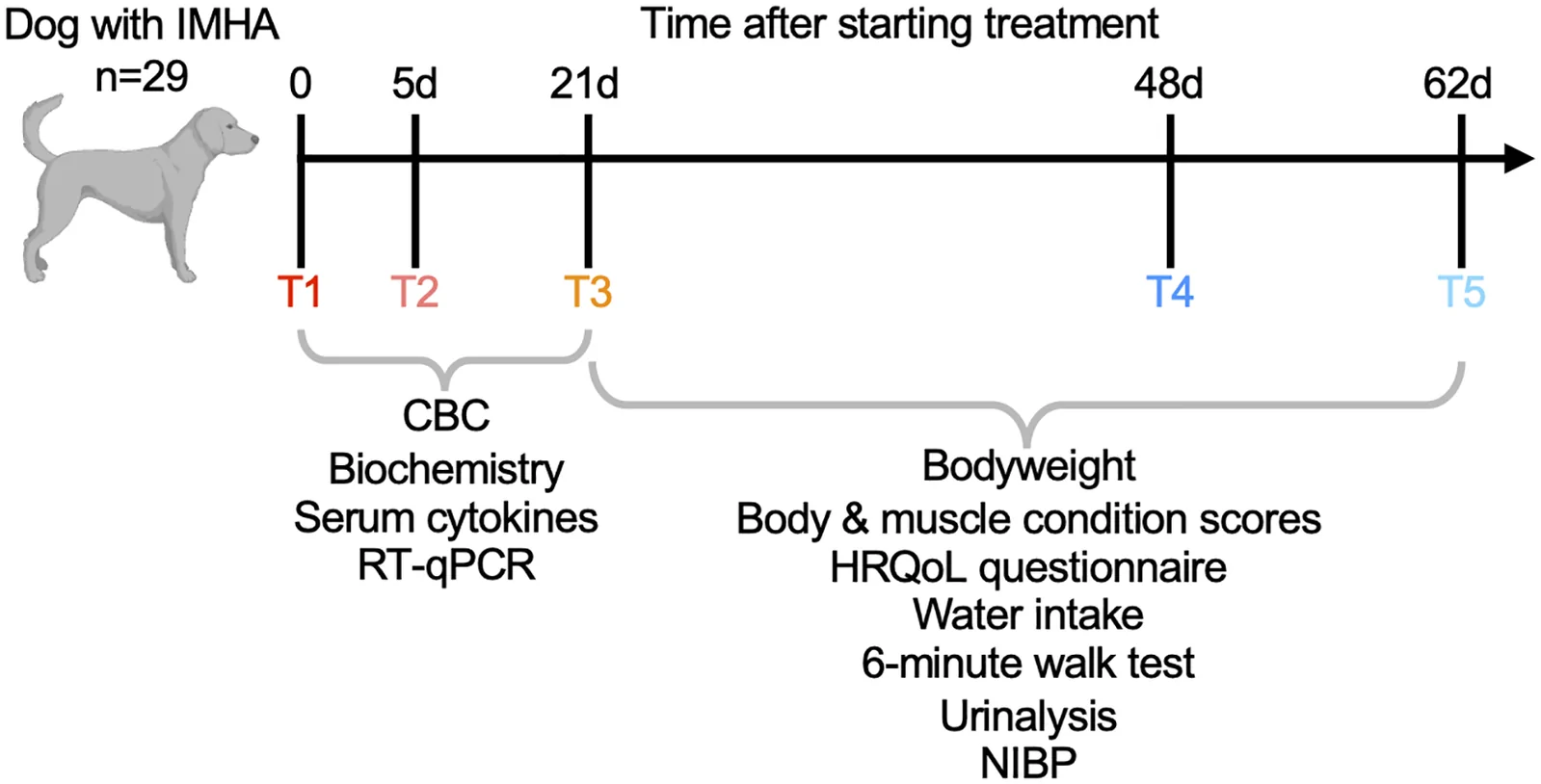
Longitudinal evaluation of dogs with IMHA. Outline showing time points for evaluation of dogs with immune-mediated hemolytic anemia (IMHA), with median times for each time after starting treatment in days (d). CBC: complete blood count, RT-qPCR: real time quantitative PCR, HRQoL: health-related quality of life, NIBP: non-invasive blood pressure.

To understand which clinical and gene expression features varied over time following onset of treatment, we clustered variables according to the extent of temporal change across 3 timepoints, representing diagnosis (T1), an early timepoint at median 5 days (inter-quartile range [IQR]: 5–6) after onset of treatment (T2), and a re-examination visit that occurred median 21 days (IQR: 19–23) after onset of treatment (T3). We identified 6 distinct clusters (**S2 Fig**), of which cluster 1 contained a single variable, PCV, that increased significantly over time, representing recovery from anemia (Fig 2a,b). Conversely, cluster 2 contained a single variable, C-reactive protein (CRP), that was initially increased as reported previously [14], but which decreased with time, likely representing amelioration of IMHA-induced inflammation. Cluster 3 contained 4 variables that showed subtle increases with time (alkaline phosphatase [ALP] and alanine aminotransferase [ALT] activity, and expression of the genes *Il10* and *Zfp36* by real time quantitative polymerase chain reactions [RT-qPCR]), whereas clusters 4 and 5 showed more complex patterns of change, with either initial increase followed by decrease (cluster 4), or decrease followed by increase (cluster 5) (Fig 2a,b). Finally, cluster 6 contained several variables that showed little change over time (**S3 Fig**). Separately, we found that all markers of RBC destruction remained abnormal in most dogs from T1 to T2, before dropping at T3 (S4 Fig). Collectively, these data show that many clinical features show significant changes with initial treatment of dogs with IMHA, making them candidates that could predict treatment response and AE severity.

**Fig 2:**
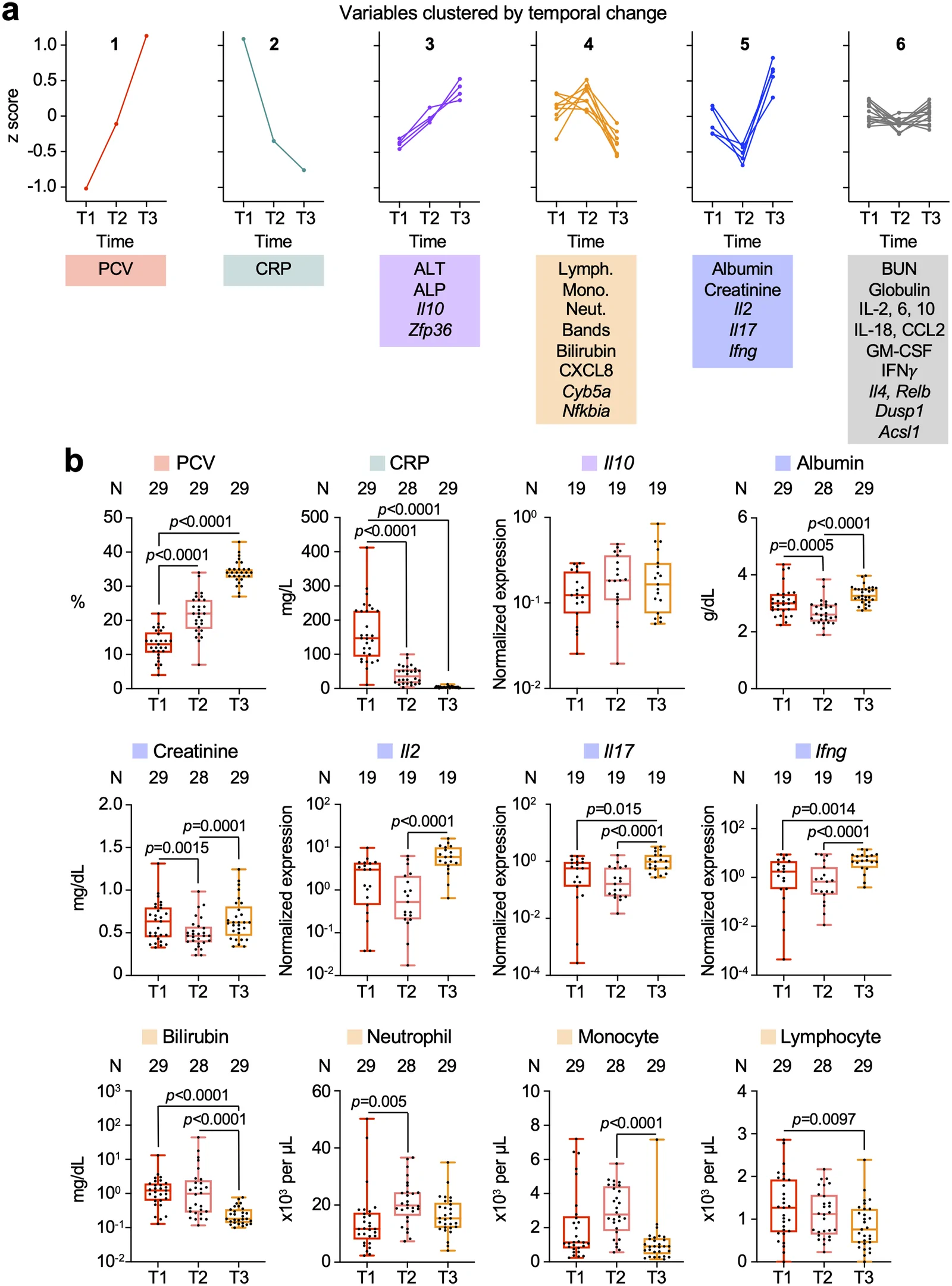
Variables show distinct patterns of temporal change. a, Changes in clinicopathological and gene expression variables in distinct temporal clusters (1-6) across times T1, T2, and T3. Points represent mean for n = 19-29 dogs for each variable, with variables assigned to each cluster shown below. **b,** Changes in indicated variables over time in dogs with IMHA. Points represent individual dogs with boxes showing median and inter-quartile range, and whiskers showing range. P values from linear mixed-effect models with pairwise estimation of marginal means. PCV: packed cell volume, CRP: C-reactive protein, ALT: alanine aminotransferase, ALP: alkaline phosphatase, Lymph.: lymphocyte, Mono.: monocyte, BUN: blood urea nitrogen, IL: interleukin, GM-CSF: granulocyte-macrophage colony stimulating factor.

### Dynamic variables are not associated with recovery from anemia

We next asked whether clinical and gene expression features that change with time might be associated with recovery from anemia in dogs with IMHA. Traditionally, outcome in IMHA has been evaluated by measuring survival, but this approach is limited because most dogs in veterinary practice are euthanized rather than dying naturally [15], and this process might be affected by owner decisions on whether to proceed with treatment. To evaluate recovery from anemia more directly, we established a new measure of outcome based on inferred time taken to return to a normal PCV. To do so, we used serial measurements of PCV from re-examination visits to fit linear or logarithmic curves for each dog before interpolating or extrapolating the time taken to achieve a PCV of 35%, which represents the lower limit of the reference interval (Fig 3a). The inferred time to reach this value varied considerably among dogs, with a median time of 26 days (IQR: 21−42) (Fig 3b), even though the average PCV increased dramatically over time (Fig 2b). In total, values were interpolated for 18 dogs (62.1%) and extrapolated for 11 (37.9%), with 3 dogs (10.3%) being assigned values of 90 days owing to long predicted times to reach a PCV of 35%. Moreover, this time correlated with duration of hospitalization (Spearman’s rho 0.46, *P* = 0.01) but not with number of blood transfusions administered (Spearman’s rho 0.25, *P* = 0.19). To evaluate the generalizability of these values, we identified an additional 35 dogs that met the same inclusion criteria for diagnosis of IMHA and had serial PCV measurements available, but which had been excluded from the study because they had incomplete residual samples (n = 16), did not follow the specified dose reduction protocol or had incomplete follow-up information (n = 5), or received a blood transfusion before presentation at the study center (n = 1). An additional 13 dogs were identified that presented to the study center after the study had closed, but which followed the same treatment regimen and schedule for follow-up visits. In this separate cohort, patterns of change in PCV were similar to those observed in dogs in the main study (**S5a, b Fig**). However, the median inferred time to reach a PCV of 35% was significantly shorter in the excluded cohort at 19 days (IQR: 15−31), albeit with considerable overlap with the main study cohort (**S5c Fig**). To determine whether dynamic clinical or gene expression features might be associated with time to reach a PCV of 35%, we performed exploratory multiple linear regression, with percentage change in dynamic variables from T2 to T1 entered as explanatory factors, while time to reach a PCV of 35% was the dependent variable. For this, we selected possible explanatory variables that (1) were in 1 of the dynamic clusters 1−4 and (2) either reflected clinical severity of disease [14,16–18] (i.e., serum bilirubin and CRP concentrations; neutrophil concentration), or were potentially involved in the aberrant autoimmune response in IMHA [19–21] (i.e., expression of genes for IL-10 and IL-17 in stimulated blood samples, see Methods: **Blood stimulation assays**). We chose not to include serum cytokine concentrations in this analysis because many analytes were not detected in many dogs. Evaluation of the model revealed that no variables were associated with time to reach a normal PCV (Fig 3c,d; **S6 Fig**). Together, these data suggest that dynamic changes in clinical features either do not predict recovery from anemia, or that this study enrolled an insufficient number of dogs with IMHA to discover these associations.

**Fig 3:**
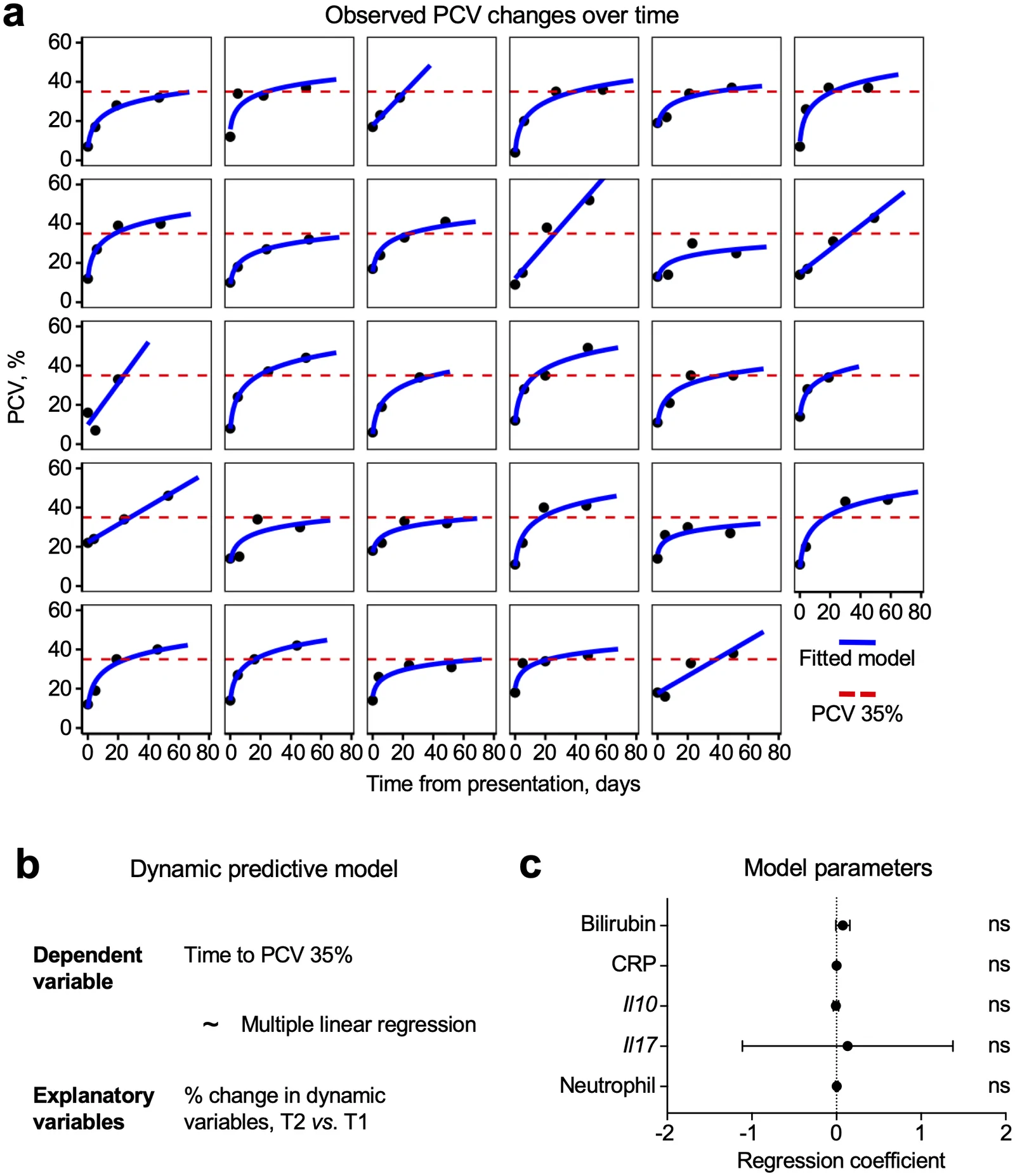
Evaluating response to treatment in dogs with IMHA. **a,** Changes in packed cell volume (PCV) over time in dogs with IMHA. Black points represent measured values for each dog, with blue line showing best fit model. Red dashed line indicates threshold of 35%. **b,** Outline for multiple linear regression model, testing potential associates of time taken to reach a PCV of 35%. **c,** Forest plot showing variables evaluated as associates of the time to reach a PCV of 35%. Points represent regression coefficients with 95% confidence intervals. P values from multiple linear regression.

### Changes in HRQoL with treatment in dogs with IMHA

To understand how health-related quality of life (HRQoL) was affected by treatment in dogs with IMHA, we used complementary quantitative and qualitative methods to evaluate changes in bodyweight and condition, muscle mass, exercise tolerance, blood pressure, and treatment-related AEs. Importantly, all dogs followed the same prednisolone dose reduction regimen, meaning that measures of AE severity could be compared across timepoints (Fig 4a), including follow-up visits at T3, T4 (median 48 days after starting treatment, IQR 48−50), and T5 (median 62 days, IQR 61−63). All dogs (n = 29) were re-presented for examination at T3, but 6 (20.7%) were lost to follow-up by T4, and 8 (27.6%) by T5 (**S1 Fig**). Among the dogs with complete records, none died or were euthanized before T5. Using available data, we found that treated dogs showed significant increases in bodyweight and body condition score over follow-up visits (Fig 4b,c), whereas muscle condition score, non-invasive blood pressure (NIBP), and exercise tolerance estimated by 6-minute walk test did not change significantly across the same times (Fig 4d,e). The water intake measured over 24 hours declined significantly from T3 to T5 (Fig 4f), and we also found that owner-reported severity of polydipsia showed the same pattern (Fig 4g). Indeed, these objective and subjective measures of water intake showed significant correlation when evaluated at T3 (Spearman’s rho 0.73, *P* = 9.98x10^-5^), suggesting owners can provide accurate estimates of water intake through their subjective estimation of polydipsia (Fig 4h). Changes in water intake were not accompanied by any significant difference in urine specific gravity (USG), with many measurements at T3 being hypersthenuric (Fig 4i), suggesting that point measurements of USG are not accurate predictors of 24-hour water consumption. Finally, a global score of HRQoL given by owners declined significantly with time (Fig 4j), indicating an overall improvement in HRQoL. Collectively, these data show that some AEs seen predominantly at early times are likely related to glucocorticoid dosage (polydipsia, owner-perceived global HRQoL), whereas others emerging at later times are probably more related to duration of glucocorticoid treatment (increased bodyweight and condition).

**Fig 4:**
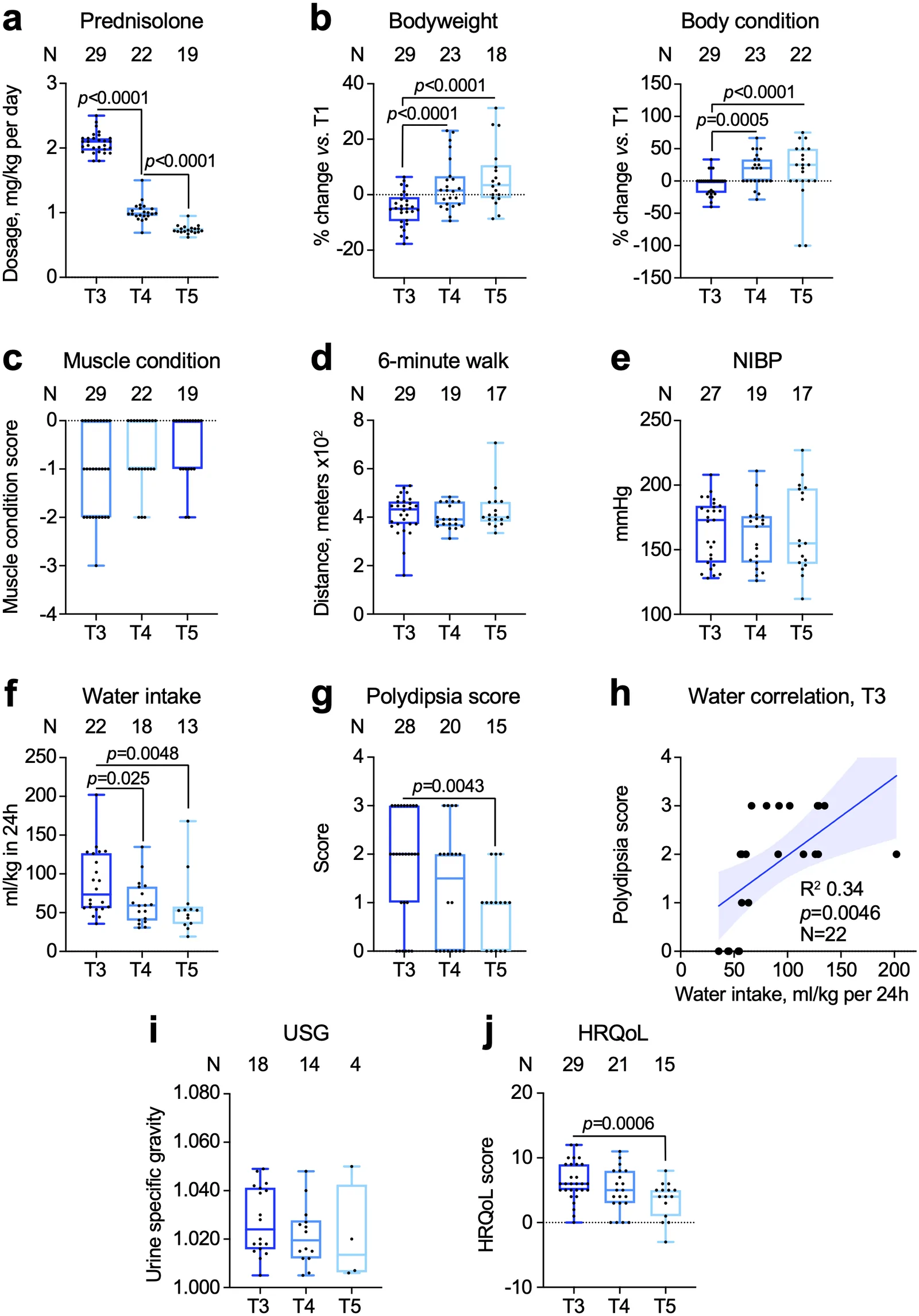
Changes in health-related quality of life in dogs with IMHA. a-g, Changes in treatment and health-related quality of life (HRQoL) parameters over time, with prednisolone dosage (a), bodyweight and body condition score (b), muscle condition score (c), 6-minute walk distance (d), non-invasive blood pressure (NIBP) measurement (e), and water intake over 24 hours (f), and owner-reported severity of polydipsia (g). Points represent indicated numbers of individual dogs, with boxes showing median and inter-quartile range, and whiskers showing range. P values from linear mixed-effect models with pairwise estimation of marginal means. **h,** Association between measured 24-hour water intake and owner-reported polydipsia score in dogs at time T3. Points represent individual dogs, line shows linear fit with shaded area showing 95% confidence interval. **i-j,** Urine specific gravity (i) and owner-reported global score for HRQoL (j) at indicated times. Points represent indicated numbers of individual dogs, with boxes showing median and inter-quartile range, and whiskers showing range. P values from linear mixed-effect models with pairwise estimation of marginal means. USG: urine specific gravity.

### Dynamic predictors of glucocorticoid-induced AEs

Polydipsia is a common glucocorticoid-associated AE that can be observed and measured readily by owners, and which is sensitive to changes in glucocorticoid dosage [9]. Using our data, we asked whether early changes in clinical and gene expression variables could be associated with severity of polydipsia, as indicated by owner-measured water intake at T3. As possible factors, we considered variables in dynamic clusters 1–4 that related to (1) metabolic responses to glucocorticoids (i.e., ALT and ALP activities), or (2) expression of glucocorticoid-regulated genes in unstimulated blood samples (i.e., *Zfp36* and *Cyb5a*) (Fig 5a, **Methods: Residual blood samples**). Using exploratory multiple linear regression (Fig 5b), we found that increasing ALP activity from time T1 to T2 was associated with greater water intake at T3 (Fig 5c,d; **S7 Fig**). Conversely, decreasing ALT activity was associated with greater water intake. Together, these data suggest that early changes in glucocorticoid-response variables might be associated with severity of AEs at later times in the treatment timeline.

**Fig 5:**
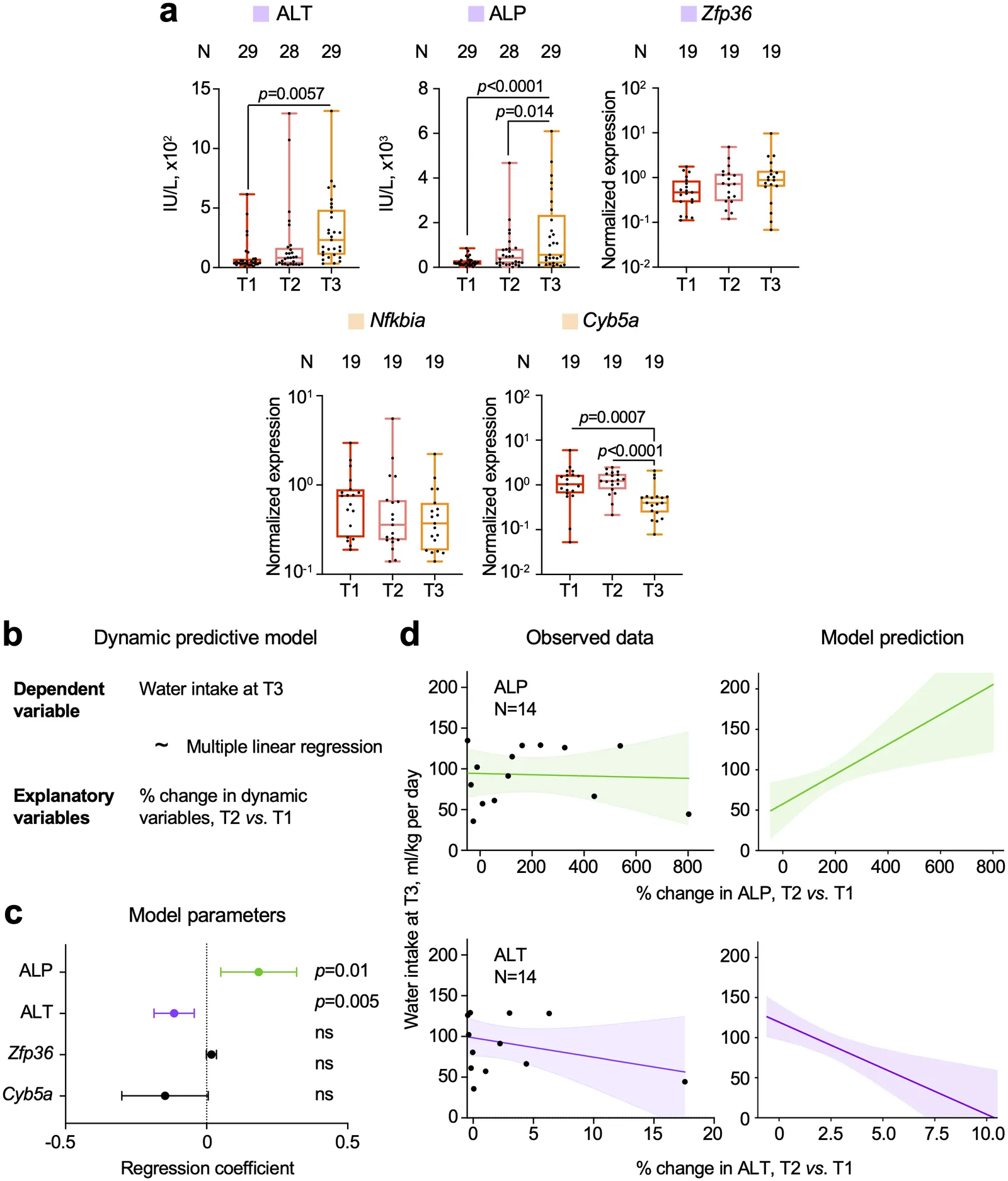
Dynamic changes predict severity of polydipsia. a, Changes in indicated variables over time. Points represent indicated numbers of individual dogs with boxes showing median and inter-quartile range, and whiskers showing range. P values from linear mixed-effect models with pairwise estimation of marginal means. ALT: alanine aminotransferase, ALP: alkaline phosphatase. **b,** Outline for multiple linear regression model, testing potential associates of water intake at time T3. **c,** Forest plot showing variables significantly associated with water intake at time T3. Points represent regression coefficients with 95% confidence intervals. P values from multiple linear regression. **d,** Observed data (left) and model predictions (right) for associations between serum ALP activity (top) and serum ALT activity (bottom) and water intake at time T3. Points represent individual dogs, lines represent linear fit (left) or model fit (right) with shaded areas showing 95% confidence interval.

## Discussion

In this study, we find that numerous clinical variables and measures of peripheral blood gene expression change after introduction of immunosuppressive treatment in dogs with IMHA. None of the evaluated dynamic variables was associated with recovery from anemia, but early increases in ALP activity were associated with more severe polydipsia at 3 weeks after starting treatment. With further validation, these data suggest that dynamic changes in the early phase of treatment could be useful clinical tools to individualize glucocorticoid regimens in dogs with IMHA.

We evaluated several dynamic variables as possible associates of time taken to return to a normal PCV value, but none was statistically significant. This suggests either that early dynamic changes are poor predictors of recovery from anemia that can occur months after onset of treatment, or that this study included insufficient numbers of dogs to reveal significant associations. When considering AEs, we found that early increases in serum ALP activity were associated with severity of polydipsia later in the treatment timeline, and this finding acts as important validation for our research approach because dogs possess a specific glucocorticoid-induced isoform of ALP that typically increases considerably when prednisolone treatment is initiated [22,23]. Together, these preliminary results indicate that dynamic trends in widely available clinical tests could offer additional insight over static measures at diagnosis that have been evaluated previously as predictors of outcome in IMHA.

Our results identify several variables that could be evaluated further to individualize treatment for dogs with IMHA, aiming to achieve effective control of hemolysis while limiting glucocorticoid-related AEs. These data contribute to a wider movement towards individualized medicine for autoimmune diseases in dogs, which also incorporates pharmacodynamic monitoring for specific drug dosages [24] and characterization of genetic polymorphisms that affect responses to treatment [25]. We envisage that early dynamic variables could be used to guide clinical therapy for dogs with IMHA (Fig 6), and further studies can now investigate whether such individualized regimens produce improved outcomes compared to standardized protocols.

**Fig 6:**
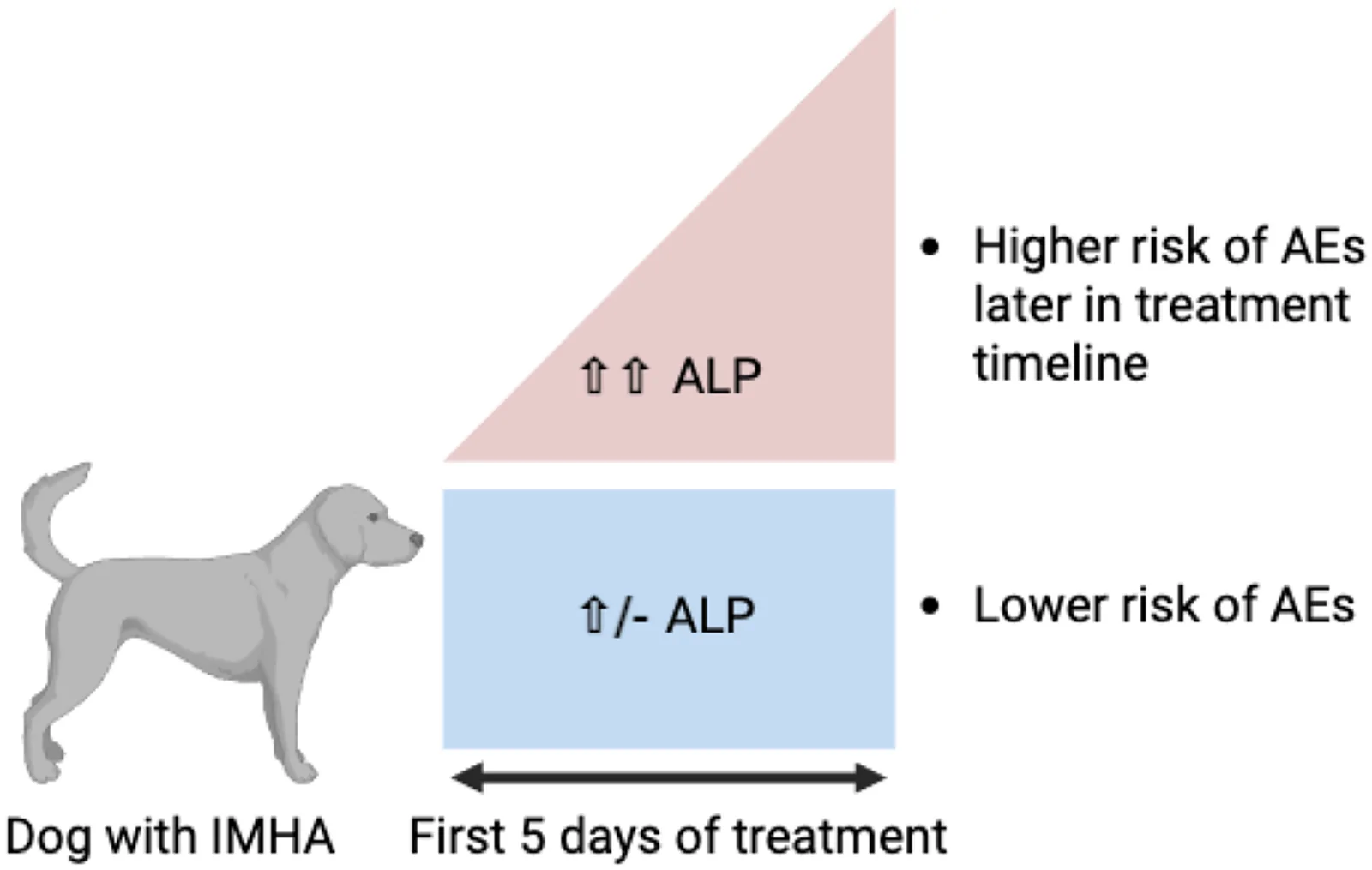
Implementing dynamic variables in clinical management. Schematic showing how evaluation of early dynamic variables could be used in clinical practice to individualize glucocorticoid treatment for dogs with IMHA, subject to validation in independent samples.

Our study has some limitations, including the relatively small number of dogs with IMHA available for study. This restricted sample size means that linear regression models could produce unstable estimates and should therefore be regarded as exploratory, with confirmation of significant associations needed in independent samples of dogs with IMHA. For HRQoL associations with dynamic variables, we evaluated associations with water intake at time T3, which is relatively early in the treatment timeline. Ideally, we would have examined similar associations at later times T4 and T5, but we opted not to do so because some cases were lost to follow-up by that time, which limited the number of dogs available for analysis. Additionally, we did not evaluate compliance of owners with instructions for water intake measurements, meaning that some estimates might not have been accurate. Similarly, we excluded several dogs from our analysis that died or were euthanized before the first follow-up visit at T3, as well as dogs that received advanced interventions like therapeutic plasmapheresis. While this permitted more comprehensive analysis of dynamic changes across multiple time points, it is possible that dogs with more severe forms of IMHA were excluded, which could have resulted in selection bias for a particular subset of dogs with IMHA and would thus decrease the applicability of our results to the wider disease population. In our analysis of AEs observed following introduction of treatment, we assumed that many changes were related to administration of prednisolone, which produces well-known changes in water intake, bodyweight, and biochemical parameters. However, since all dogs were also treated with azathioprine, clopidogrel, and omeprazole in the early stages of treatment, we cannot exclude the possibility that some AEs were related to these drugs, which might limit the applicability of our conclusions to dogs treated with prednisolone alone.

In conclusion, we find that many clinicopathological and gene expression variables show temporal changes during treatment of dogs with IMHA, with early increases in serum ALP activity being associated with later severity of polydipsia. This parameter is therefore a key candidate for further investigations of individualization of immunosuppressive therapy for dogs with IMHA.

## Materials and methods

### Study design

We conducted a prospective, observational study at a university referral hospital, enrolling client-owned dogs with non-associative IMHA that fulfilled the “diagnostic” definition given by the ACVIM consensus statement on diagnosis of IMHA [26]. All dogs in this study were pet animals, which were brought to the veterinary hospital for treatment of naturally occurring disease; no dogs in this study were bred for research purposes, no clinical interventions were conducted that were not indicated for the benefit of the individual animal, all treatment interventions reflected common clinical practice for management of IMHA in dogs, and all experimental procedures were performed on residual blood samples. Therefore, no humane endpoints were defined for this study, and this study design was approved by the Royal Veterinary College ethical review committee (reference number URN 2024-08-2). While hospitalized at the study institution, all dogs received veterinary care according to principles of good veterinary practice, which was provided by qualified veterinarians and veterinary nurses registered with the Royal College of Veterinary Surgeons, UK. This care consisted of physical examinations performed at least twice daily, administration of drugs on 24-hour schedules, provision of appropriate meals at least twice daily, and brief periods of exercise outside at least every 4 hours. Anesthesia and analgesia were not required for blood sampling, but dogs considered to show any signs of discomfort while hospitalized were treated with methadone (0.1-0.4 mg/kg intravenously every 4 hours) or buprenorphine (0.01-0.02 mg/kg intravenously every 6–8 hours). After discharge from the study institution, dogs were presented for follow-up examinations according to the schedule shown in Fig 1, when they were examined by a veterinarian. Owners of dogs gave informed consent for enrolment of their dogs in the study. Dogs were enrolled in the study until 8 weeks after starting treatment for IMHA. After this time, their clinical care continued under the direction of the attending veterinarian. Some dogs in the study died of natural causes or were euthanized by a veterinarian within 8 weeks of starting treatment for IMHA owing to clinical deterioration. All dogs were enrolled in the study and treated with the intention of survival; therefore, euthanasia was not a planned endpoint, and no criteria were specified for euthanasia for the purpose of this study. Decisions about euthanasia were made by owners of individual dogs in consultation with the attending veterinarian. Where dogs were euthanized, this was performed by intravenous injection of pentobarbitone (200 mg/ml solution administered at ~1 ml/kg through an intravenous cannula).

### Inclusion criteria

Dogs presented to the study center were enrolled if they fulfilled all of the following criteria:

- Anemic, with a hematocrit or PCV < 35%
- At least one of the following measures of anti-RBC antibodies:
  - Positive point-of-care direct antiglobulin test (Easy QuickTest DAT, Alvedia), performed according to the manufacturer’s instructions.
  - Agglutination of RBCs after dilution 49:1 in saline, confirmed microscopically.
  - Moderate or marked spherocytosis on examination of a fresh blood smear by a board-certified clinical pathologist.
- At least one of the following indicators of hemolysis:
  - Hemolysed plasma
  - Hemoglobinuria and/or bilirubinuria
  - Hyperbilirubinemia
- No underlying cause of IMHA detected after reviewing a biochemical profile, results of imaging of the thorax and abdomen, serological tests for *Anaplasma spp.*, *Ehrlichia spp.*, *Borrelia burgdorferi*, and antigen testing for *Dirofilaria immitis* (4DX SNAP test, IDEXX) if there was a history of travel outside the UK, and urinalysis.
- Did not receive immunosuppressive treatment for more than 24 hours before presentation.
- No previous episodes of IMHA in the clinical history.
- No history of vaccination in the 2 weeks before onset of clinical signs.

### Exclusion criteria

Dogs were excluded from the study if they died or were euthanized before the follow-up visit that was scheduled at 2 weeks after discharge from the study institution, or if they deviated from the recommended treatment protocol or were lost to follow-up before that time. Dogs were also not included if they received blood products before presentation at the study institution, or if they received glucocorticoids for more than 24 hours before this time. Dogs were excluded if they required any treatment not specified in the study protocol, such as other immunosuppressive drugs, therapeutic plasma exchange, or heparins. Finally, dogs were excluded if incomplete residual samples were available at times T1 and T2.

### Residual blood samples

Blood was collected from dogs enrolled in the study for diagnostic purposes, and any residual volume was made available to the study investigators after diagnostic tests were completed. Residual serum was frozen at −80°C in 50 μl aliquots until cytokine assays were completed in batches. Residual EDTA-anticoagulated blood (0.3-0.5 ml) was mixed with RNAlater solution (1.3 ml, ThermoFisher Scientific, AM7021) then frozen at −80°C until RNA was extracted in batches. Residual citrate-anticoagulated blood samples were used within 24 hours for stimulation assays, as described below.

### Blood stimulation assays

Citrate-anticoagulated blood was transferred to a 15 ml tube and centrifuged at 1,000 *g* for 5 minutes. The supernatant was discarded, and cells were re-suspended in RPMI-1640 medium (Lonza, BE12-702F) containing 20% fetal bovine serum (Sigma-Aldrich, F9665), 1.25 μg/ml phorbol 12-myristate-13-acetate (PMA, Cambridge Bioscience, 10008014), and 1.2 μg/ml ionomycin (Sigma-Aldrich, IO634) in a final volume of 1 ml. This solution was transferred to a 24-well plate (Corning, 353047) and incubated for 3 hours at 37°C and 5% CO_2_ in a tissue culture incubator. The contents of each well were subsequently transferred to a 1.5 ml tube and centrifuged at 11,300 *g* for 5 minutes. The supernatant was discarded, and the cell pellet was re-suspended in 1.3 ml RNAlater solution, before tubes were frozen at −80°C until RNA was extracted.

### RT-qPCR reactions

Real time quantitative PCR (RT-qPCR) reactions were performed according to published guidelines [27]. RNA was extracted from stimulated samples or from EDTA-anticoagulated blood in batches using the RiboPure RNA Purification Kit (ThermoFisher Scientific, AM1928) according to the manufacturer’s instructions. The concentration of extracted RNA was measured using a NanoDrop Lite Plus spectrophotometer (ThermoFisher Scientific), and 10 ng was used as input for synthesis of complementary (c)DNA using the High-Capacity cDNA Reverse Transcription Kit (ThermoFisher Scientific, 4368814). Final RT-qPCR reactions were then conducted in 96 well plates (ThermoFisher Scientific, AB0800W), using 10 μl Precision FAST qPCR mastermix (PrimerDesign), 1 μl Taqman primers (listed in **S3 Table**), 5 μl molecular water (Corning, 46–000-CI), and 4 μl cDNA solution. Reactions for each gene were conducted in duplicate for each sample, and plates were run in a CFX96 real time detection instrument (Bio-Rad) with data collection in the FAM channel and with the following run cycle parameters: cycle 1: 95^o^C for 2 minutes, cycles 2–46: 95^o^C for 5 seconds, 60^o^C for 20 seconds with data collection. Reactions with failed amplification or with Cq values >40 were excluded from analysis. Reference genes were selected by running a geNORM kit (PrimerDesign) to evaluate 5 different genes for stable expression in an initial set of paired samples collected at steady state and after 2 weeks of prednisolone treatment in 8 dogs. Results of this analysis indicated that a combination of 2 genes (*Rpl13a* and *Sdha*) provided the most stable reference (**S8 Fig**), aligning with a previous study confirming the stability of *Rpl* gene expression in canine tissues [28]. Expression values for query genes were calculated using the formula:


$$\begin{array}{c} Relativeexpression={\text{2}}^{-\left(queryCq-\sqrt{Rpl\text{1}\text{3}aCq\times SdhaCq}\right)} \end{array}$$

### Cytokine assays

Concentrations of 9 cytokines (granulocyte macrophage colony stimulating factor [GM-CSF], tumor necrosis factor [TNF]-α, monocyte chemoattractant protein [MCP]-1 also known as CCL2, interferon [IFN]-γ, CXCL8, and interleukins [IL]-2, IL-6, IL-10, and IL-18 were measured in serum using the MILLIPLEX Canine Cytokine/Chemokine Magnetic Bead Assay (Merck Millipore) according to the manufacturer’s instructions. Serum samples were thawed and diluted 1:2 in assay buffer for measurement, and expression levels were evaluated on a Magpix instrument (Luminex). All cytokine measurements were performed in duplicate for each dog, and samples with no detectable cytokine were assigned a value representing the lower limit of detection provided by the manufacturer.

### Treatment and follow-up schedule

All dogs followed a standardized treatment regimen, which began with administration of prednisolone at 3 mg/kg *per os* [PO] once daily with azathioprine (50 mg/m^2^ PO every other day, or 50–75 mg/m^2^ every third day in dogs <10 kg bodyweight), clopidogrel (2 mg/kg PO once daily), and omeprazole (1 mg/kg PO once daily). Dogs that could not tolerate oral medications were initially treated with dexamethasone sodium succinate administered intravenously (0.4 mg/kg once daily). After this, dogs followed the dose reduction schedule outlined in Table 1.

**Table 1:** Outline for treatment and follow-up visits for dogs in the study.

Time in treatment timeline	Prednisolone dosage (mg/kg per day)	Azathioprine dosage (mg/m^2^ every 2^nd^ day)	Clopidogrel dosage (mg/kg per day)	Omeprazole dosage (mg/kg per day)	Clinical monitoring tests
Start treatment	3	50	2-4	1	CBC, BC, imaging, DAT, UA
Discharge to 1 week visit	2	50	2-4	0	PCV/TP
1 week to 2 week visit	2	50	2-4	0	CBC, BC, DAT, UA
2 week to 4 week visit	1.5	50	2-4	0	PCV/TP
4 week to 6 week visit	1	50	2-4	0	CBC, BC, DAT
6 week to 8 week visit	0.75	50	2-4	0	CBC
After 8 weeks	Clinician discretion				

BC: biochemical profile, CBC: complete blood count, DAT: direct antiglobulin test, PCV: packed cell volume, TP: total protein, UA: urinalysis. For dogs under 10 kg, azathioprine was dosed at 50–75 mg/m^2^ every third day.

### Health-related quality of life assessment

To evaluate HRQoL during treatment, a questionnaire was designed for completion by dog owners. The questionnaire consisted of 11 questions on specific clinical signs or features of glucocorticoid treatment scored on a 7-point Likert scale, paired with a question on the perceived importance of these features for the dog scored on a 4-point Likert scale, as well as a global score of HRQoL. The questionnaire was circulated to veterinarians and dog owners in the study institution, and their feedback was used to finalize the text, which is shown in S1 File Information. To provide an objective measure of water intake, we provided dog owners with a graduated measuring jug upon enrolment to the trial, along with instructions on how to measure water intake over a period of 24 hours. Owners were asked to complete water intake measurements in the few days before or after re-examination appointments conducted at times T3, T4, and T5, and the bodyweights of the dogs recorded at those visits were used to calculate water intake per kilogram. The study investigators did not conduct any specific monitoring to ensure that owners were complying with instructions for water intake measurement. To evaluate exercise tolerance, we performed 6-minute walk tests as described previously [29] by measuring the distance that dogs could walk at a comfortable walking pace for 6 minutes. For this, dogs were walked at a comfortable walking pace back and forth along the same corridor in the veterinary hospital, in which length had been measured previously. The number of complete laps of the corridor was recorded, along with a measurement of distance travelled in any final incomplete lap, within a time of 6 minutes. Non-invasive measurement of blood pressure (NIBP) was performed using a Doppler device (Doppler Flow Detector Model 811-B, Parks Medical Electronics) according to published guidelines [30]. Body condition score and muscle condition score were evaluated by veterinarians at re-evaluation visits using established scales [31,32].

### Statistical analysis

All statistical analyses were completed with R (version 4.5.2) and GraphPad Prism (version 10.6.1). Correlations between variables were evaluated by calculating Spearman’s rho. Changes in variables over time were evaluated by fitting linear mixed-effect models, using time point as a fixed effect and dog identity as a random intercept to account for repeated measures. Models were fitted using the *lmer* function in the R package *lme4* (version 2.0−1). Where significant main effects were observed, pairwise comparisons were performed between different time points by calculation of estimated marginal means using the *emmeans* function in the *emmeans* package (version 2.0.3). Model assumptions were verified by visual inspection of plotted residuals against fitted values and in quantile-quantile plots, generated with the *qqnorm* function in the R package *stats* (version 3.6.2). To evaluate patterns of temporal change in clinical and gene expression variables, all variables were standardized to the same scale by calculation of z scores across time points, and mean z score values for each time point were then clustered hierarchically using the *hclust* function in the R package *stats*. The dendrogram was cut to identify k = 6 clusters.

To derive a new measure of IMHA outcome, time to reach a PCV of 35%, we used repeated PCV measurements from each dog to fit both linear and logarithmic curves using the *lm* function in the R *stats* package. For each dog, we compared r^2^ values between the linear and logarithmic models before selecting the model with the better fit. From these, we interpolated or extrapolated the time point at which a PCV value of 35% would be reached in each dog. For dogs with inferred values >90 days, we set the value to 90 days to prevent extreme values from distorting subsequent analysis. To identify variables associated with either time to reach a PCV of 35% or with measured water intake, we used multiple linear regression, applied with the *lm* function. Dynamic explanatory variables were entered as percentage changes from time T1 to T2. Predicted relationships between explanatory and outcome variables were generated with the *ggpredict* function from the R package *ggeffects* (version 2.3.2). All data generated in this study are available in S2 File.

## Supporting information

S1 Fig Selection of study cases. Flow chart showing reasons for exclusion of cases considered for inclusion in this study, along with reasons for loss-to-follow-up for enrolled cases.
(TIFF)

S2 Fig Clustering of variables according to temporal change. Dendrogram showing relationships among variables according to patterns of temporal change of scaled z scores across times T1, T2, and T3. Boxes and numbers indicate selection of k = 6 clusters.
(TIFF)

S3 Fig Variables showing little temporal change. Changes in indicated variables over time. Points represent individual dogs with boxes showing median and inter-quartile range, and whiskers showing range. *P values* from linear mixed-effect models with pairwise estimation of marginal means. BUN: blood urea nitrogen.
(TIFF)

S4 Fig Changes in red blood cell destruction metrics. Line plots showing change in proportion of dogs with indicated features of immune-mediated red blood cell destruction. Points represent proportions for n = 29 dogs, with shaded areas showing 95% confidence intervals.
(TIFF)

S5 Fig Changes in packed cell volume in dogs with IMHA. a, Scatter plots showing packed cell volume (PCV) measurements over time in an additional cohort of dogs with IMHA that were excluded from the main study. Points represent individual PCV measurements, with blue lines showed fitted curves, and red dashed lines showing PCV value of 35%. b, Overall models for all dogs in either main study (left, n = 29) or excluded (right, n = 35) cohorts, with fitted models. Blue dashed lines indicate the inferred time to reach a PCV of 35% in all dogs in each cohort. Points represent individual PCV measurements in all dogs in each group. c, Boxplot showing inferred times to reach a PCV of 35% in main study and excluded cohorts. Points represent individual dogs with median and inter-quartile range. Whiskers show range. *P value* from Mann-Whitney U test.
(TIFF)

S6 Fig Model parameters for associations with recovery from anemia. Data showing complete model coefficients, model parameters, and diagnostic plots for associations between indicated explanatory variables and predicted time to reach a PCV of 35%. Q-Q: quantile-quantile.
(TIFF)

S7 Fig Model parameters for associations with water intake. Data showing complete model coefficients, model parameters, and diagnostic plots for associations between indicated explanatory variables and severity of polydipsia at time T3. Q-Q: quantile-quantile.
(TIFF)

S8 Fig Selection of stable reference genes for RT-qPCR. Line plot showing stability of indicated candidate reference genes in n = 10 pilot samples from canine peripheral blood.
(TIFF)

S1 Table Demographic features of dogs with IMHA. (DOCX)

S2 Table Clinical features of dogs with IMHA at presentation. (DOCX)

S3 Table Primers used for real-time quantitative polymerase chain reactions. (DOCX)

S1 File Transcript of questionnaire to assess HRQoL following onset of treatment for IMHA. (PDF)

S2 File Complete dataset for data collected in this study. a, Complete dataset for cases in the main study (n = 29). b, Changes in PCV values for cases in the excluded cohort (n = 35).
(XLSX)
