# Supplementary material for: Dynamic changes in clinicopathological variables and gene expression in dogs with immune-mediated hemolytic anemia
Source: PLoS One. 2026 Oct 28;21(10):e0359710. doi: 10.1371/journal.pone.0359710 (PMC13716216; doi:10.1371/journal.pone.0359710)
Supplement: Supplementary file 1 [file pone.0359710.s012.pdf]

Thank you for taking the time to complete this questionnaire.

This questionnaire consists of several questions about different aspects of your dog's behaviour, with associated questions asking how important you think any changes are for your dog's wellbeing.

The information you provide is intended to help us understand that changes that occur in dogs receiving steroids so that we can improve the care we give them in future. Use of this questionnaire has been approved by Clinical Research Ethical Review Board at the Royal Veterinary College, University of London.

The information you provide will be used for research purposes but we will not use or store any personal information. Similarly, the information you provide will not be passed to any of the clinicians treating your dog, so please contact the Queen Mother Hospital for Animals directly if you have any concerns about your dog.

Quality of Life of Dogs receiving Steroids

1. Please enter the study number we sent you in the e-mail or letter with the link to this questionnaire

2. Please tell us the current dose of the steroids your dog is receiving (for example: 5 mg tablet once daily)

If your dog is receiving multiple drugs, the steroid will be marked'Prednisolone' or 'Prednicare'.

3. Please tell us, to the nearest week, how long your dog has been receiving steroids during the current course. (If your dogs has received steroids in the past but had a break in between then and the current course, please just tell us how long he/she has been treated in the current course).

Quality of Life of Dogs receiving Steroids

4. Compared to his/her normal behaviour, how much has your dog been drinking over the past week?

Much less than normal			Normal amount			Much more than normal
○	○	○	○	○	○	○

5. Do you think this is having any effect on your dog's wellbeing?

Not at all	A minor effect	A moderate effect	A major effect
○	○	○	○

6. Compared to his/her normal behaviour, how much has your dog been urinating over the past week?

Much less than normal			Normal amount			Much more than normal
○	○	○	○	○	○	○

7. Do you think this is having any effect on your dog's wellbeing?

Not at all	A minor effect	A moderate effect	A major effect
○	○	○	○

8. Compared to his/her normal behaviour, how hungry has your dog been over the past week?

Much less hungry than normal			Normal appetite			Much hungrier than normal
○	○	○	○	○	○	○

9. Do you think this is having any effect on your dog's wellbeing?

Not at all	A minor effect	A moderate effect	A major effect
○	○	○	○

10. Compared to his/her normal behaviour, how much has your dog been defaecating (pooing) over the past week?

Defaecating much less than normal			Defaecating normal amount			Defaecating much more than normal
○	○	○	○	○	○	○

11. Do you think this is having any effect on your dog's wellbeing?

Not at all	A minor effect	A moderate effect	A major effect
○	○	○	○

Quality of Life of Dogs receiving Steroids

12. Compared to his/her normal behaviour, how enthusiastic has your dog been to go outside for exercise over the past week?

Much less enthusiastic than normal			Normal level of enthusiasm			Much more enthusiastic than normal
○	○	○	○	○	○	○

13. Do you think this is having any effect on your dog's wellbeing?

Not at all	A minor effect	A moderate effect	A major effect
○	○	○	○

14. Compared to his/her normal behaviour, how much has your dog been panting over the past week?

Panting much less than normal			Panting a normal amount			Panting much more than normal
○	○	○	○	○	○	○

15. Do you think this is having any effect on your dog's wellbeing?

Not at all	A minor effect	A moderate effect	A major effect
○	○	○	○

16. Compared to his/her normal behaviour, how energetic has your dog been at home over the past week?

Much less energetic than normal			Normal amount of energy			Much more energetic than normal
○	○	○	○	○	○	○

17. Do you think this is having any impact on your dog's wellbeing?

Not at all	A minor effect	A moderate effect	A major effect
○	○	○	○

18. Compared to his/her normal behaviour, how much has your dog been playing at home over the past week?

Playing much less than normal			Playing a normal amount			Playing much more than normal
○	○	○	○	○	○	○

19. Do you think this is having any effect on your dog's wellbeing?

Not at all	A minor effect	A moderate effect	A major effect
○	○	○	○

20. Compared to his/her normal behaviour, how friendly has your dog been towards other pets and people at home over the past week?

Much less friendly than normal			Normal behaviour towards them			Much more friendly than normal
○	○	○	○	○	○	○

21. Do you think this is having any effect on your dog's wellbeing?

Not at all	A minor effect	A moderate effect	A major effect
○	○	○	○

22. Compared to his/her normal behaviour, how confident has your dog been over the past week?

Much more nervous than normal			Normal level of confidence			Much more confident than normal
○	○	○	○	○	○	○

23. Do you think this is having any effect on your dog's wellbeing?

Not at all	A minor effect	A moderate effect	A major effect
○	○	○	○

24. Compared to his/her normal behaviour, how much has your dog wanted to be with you over the past week?

Following you and seeking your attention much less than normal			Normal level of following and attention seeking			Following you and seeking your attention much more than normal
○	○	○	○	○	○	○

25. Do you think this is having any effect on your dog's wellbeing?

Not at all	A minor effect	A moderate effect	A major effect
○	○	○	○

Quality of Life of Dogs receiving Steroids

26. Considering all aspects of your dog's behaviour, how would you rate his/her quality of life at the moment?

Very bad			Neither good nor bad			Very good
○	○	○	○	○	○	○

Quality of Life of Dogs receiving Steroids

27. Thank you for completing this questionnaire.

Is there something else you would like to tell us about your dog?

Did you think any of the questions were difficult to answer, or we did we miss anything important?

Please let us know in the box below.
