# Supplementary material for: Dynamic changes in clinicopathological variables and gene expression in dogs with immune-mediated hemolytic anemia
Source: PLoS One. 2026 Oct 28;21(10):e0359710. doi: 10.1371/journal.pone.0359710 (PMC13716216; doi:10.1371/journal.pone.0359710)
Supplement: Supplementary file 2 [file pone.0359710.s009.docx]

**Supplementary Table 1**: Demographic features of dogs with IMHA.

| **Parameter** |  | **Value** |
| --- | --- | --- |
| Age (months) |  |  |
|  | Median, IQR | 100 (60-115) |
| Sex (N, %) |  |  |
|  | Female intact | 1 (3.4%) |
|  | Female neutered | 18 (62.1%) |
|  | Male intact | 1 (3.4%) |
|  | Male neutered | 9 (31.0%) |
| Breeds (N, %) |  |  |
|  | Cross breed | 7 (24.1%) |
|  | Cocker spaniel | 2 (6.9%) |
|  | Springer spaniel | 2 (6.9%) |
|  | West Highland white terrier | 2 (6.9%) |
|  | Labrador retriever | 2 (6.9%) |
|  | Bichon frise | 2 (6.9%) |
|  | Maltese terrier | 2 (6.9%) |
|  | Other | 10 (34.5%) |
