# Supplementary material for: Dynamic changes in clinicopathological variables and gene expression in dogs with immune-mediated hemolytic anemia
Source: PLoS One. 2026 Oct 28;21(10):e0359710. doi: 10.1371/journal.pone.0359710 (PMC13716216; doi:10.1371/journal.pone.0359710)
Supplement: Supplementary file 3 [file pone.0359710.s010.docx]

**Supplementary Table 2**: Clinical features of dogs with IMHA at presentation (n=29).

| **Parameter** | **Data type** | **Value** | **Reference interval** |
| --- | --- | --- | --- |
| Positive direct antiglobulin test | N (%) | 18 (62.1%) |  |
| Positive saline agglutination test | N (%) | 26 (89.7%) |  |
| Spherocytosis | N (%) | 23 (79.3%) |  |
| Packed cell volume (%) | Median (IQR) | 13 (11-16) | 35-55 |
| Neutrophil count (x10^3^/μl) | Median (IQR) | 11.7 (9.1-16.9) | 3.0-11.5 |
| Monocyte count (x10^3^/μl) | Median (IQR) | 1.1 (0.8-2.6) | 0.15-1.5 |
| Lymphocyte count (x10^3^/μl) | Median (IQR) | 1.3 (0.7-1.9) | 1.0-4.8 |
| Reticulocytes (%) | Median (IQR) | 7.4 (4.6-15.8) |  |
| Serum albumin (g/dL) | Median (IQR) | 3.0 (2.8-3.3) | 2.6-3.8 |
| Serum globulin (g/dL) | Median (IQR) | 3.6 (3.3-3.9) | 2.3-4.2 |
| Serum bilirubin (mg/dL) | Median (IQR) | 1.22 (0.62-1.94) | 0.01-0.25 |
| Serum creatinine (mg/dL) | Median (IQR) | 0.63 (0.46-0.79) | 0.22-1.63 |
| Serum blood urea nitrogen (mg/dL) | Median (IQR) | 22.5 (14.0-31.5) | 8.7-28.3 |
| Serum ALT activity (IU/L) | Median (IQR) | 41.1 (29.3-72.9) | 19.8-124.0 |
| Serum ALP activity (IU/L) | Median (IQR) | 190 (113-315) | 0-130 |
| Serum C-reactive protein (mg/L) | Median (IQR) | 147.1 (99.8-226.9) |  |
