# Supplementary material for: Dynamic changes in clinicopathological variables and gene expression in dogs with immune-mediated hemolytic anemia
Source: PLoS One. 2026 Oct 28;21(10):e0359710. doi: 10.1371/journal.pone.0359710 (PMC13716216; doi:10.1371/journal.pone.0359710)
Supplement: Supplementary file 4 [file pone.0359710.s011.docx]

**Supplementary Table 3**: Primers used for real-time quantitative polymerase chain reactions (RT-qPCR).

| **Gene** | **Group** | **ThermoFisher Scientific Taqman primer** |
| --- | --- | --- |
| *Il2* | T cell cytokine | Cf02623317_m1 |
| *Il4* | T cell cytokine | Cf02623112_m1 |
| *Il10* | T cell cytokine | Cf02624265_m1 |
| *Il17* | T cell cytokine | Cf03993800_m1 |
| *Ifng* | T cell cytokine | Cf02623316_m1 |
| *Dusp1* | Glucocorticoid-regulated | Cf02639271_g1 |
| *Cyb5a* | Glucocorticoid-regulated | Cf02668330_g1 |
| *Nfkbia* | Glucocorticoid-regulated | Cf02741714_m1 |
| *Zfp36* | Glucocorticoid-regulated | Cf02648767_g1 |
| *Acsl1* | Glucocorticoid-regulated | Cf02696419_m1 |
| *Relb* | Glucocorticoid-regulated | Cf02699180_m1 |
| *Pdk4* | Glucocorticoid-regulated | Cf02700632_m1 |
| *Sdha* | Reference | Cf02664981_m1 |
| *Rpl13a* | Reference | Cf04947268_gH |
| *Gapdh* | Reference | Cf04419463_gH |
| *Hprt1* | Reference | Cf02690456_g1 |
| *B2m* | Reference | Cf02659077_m1 |
