# Supplementary material for: Dynamic changes in clinicopathological variables and gene expression in dogs with immune-mediated hemolytic anemia
Source: PLoS One. 2026 Oct 28;21(10):e0359710. doi: 10.1371/journal.pone.0359710 (PMC13716216; doi:10.1371/journal.pone.0359710)
Supplement: Supplementary file 5 [file pone.0359710.s015.docx]

PONE-D-26-22460

Dynamic changes in clinicopathological variables and gene expression predict recovery from anemia and treatment-related adverse effects in dogs with immune-mediated hemolytic anemia

PLOS One

Dear Dr. Swann,

Thank you for submitting your manuscript to PLOS ONE. After careful consideration, we feel that it has merit but does not fully meet PLOS ONE’s publication criteria as it currently stands. Therefore, we invite you to submit a revised version of the manuscript that addresses the points raised during the review process.

Please submit your revised manuscript by Sep 04 2026 11:59PM. If you will need more time than this to complete your revisions, please reply to this message or contact the journal office at [plosone@plos.org](mailto:plosone@plos.org). When you're ready to submit your revision, log on to https://www.editorialmanager.com/pone/ and select the 'Submissions Needing Revision' folder to locate your manuscript file.

Please include the following items when submitting your revised manuscript:

- A letter that responds to each point raised by the academic editor and reviewer(s). You should upload this letter as a separate file labeled 'Response to Reviewers'.
- A marked-up copy of your manuscript that highlights changes made to the original version. You should upload this as a separate file labeled 'Revised Manuscript with Track Changes'.
- An unmarked version of your revised paper without tracked changes. You should upload this as a separate file labeled 'Manuscript'.

If you would like to make changes to your financial disclosure, please include your updated statement in your cover letter. Guidelines for resubmitting your figure files are available below the reviewer comments at the end of this letter.

If applicable, we recommend that you deposit your laboratory protocols in protocols.io to enhance the reproducibility of your results. Protocols.io assigns your protocol its own identifier (DOI) so that it can be cited independently in the future. For instructions see: [https://journals.plos.org/plosone/s/submission-guidelines#loc-laboratory-protocols](https://nam02.safelinks.protection.outlook.com/?url=https%3A%2F%2Ftrack.editorialmanager.com%2FCL0%2Fhttps%3A%252F%252Fjournals.plos.org%252Fplosone%252Fs%252Fsubmission-guidelines%2523loc-laboratory-protocols%2F1%2F010f019f3a88c9bf-b8517ac3-d107-4c70-b9d9-95d8bb3339ee-000000%2FbWZmhSXjtBHovku7LiO_4wb5mpZTJscDsI7_UR1AUrc%3D258&data=05%7C02%7Cjs5895%40cumc.columbia.edu%7C85eae93336524483ade108dedbd477a5%7Cb0002a9b0017404d97dc3d3bab09be81%7C0%7C0%7C639189903066361354%7CUnknown%7CTWFpbGZsb3d8eyJFbXB0eU1hcGkiOnRydWUsIlYiOiIwLjAuMDAwMCIsIlAiOiJXaW4zMiIsIkFOIjoiTWFpbCIsIldUIjoyfQ%3D%3D%7C0%7C%7C%7C&sdata=WGccefdgZEf5UmZURWCHHFU8VW0SpTqcT%2F1d5AE7VP0%3D&reserved=0). Additionally, PLOS ONE offers an option for publishing peer-reviewed Lab Protocol articles, which describe protocols hosted on protocols.io. Read more information on sharing protocols at [https://plos.org/protocols?utm_medium=editorial-email&utm_source=authorletters&utm_campaign=protocols](https://nam02.safelinks.protection.outlook.com/?url=https%3A%2F%2Ftrack.editorialmanager.com%2FCL0%2Fhttps%3A%252F%252Fplos.org%252Fprotocols%253Futm_medium%3Deditorial-email%2526utm_source%3Dauthorletters%2526utm_campaign%3Dprotocols%2F1%2F010f019f3a88c9bf-b8517ac3-d107-4c70-b9d9-95d8bb3339ee-000000%2FkL7mQ4cUlLL_Khumtw1uXxkJ1_lBRQ4vsBsH84r_uiw%3D258&data=05%7C02%7Cjs5895%40cumc.columbia.edu%7C85eae93336524483ade108dedbd477a5%7Cb0002a9b0017404d97dc3d3bab09be81%7C0%7C0%7C639189903066390710%7CUnknown%7CTWFpbGZsb3d8eyJFbXB0eU1hcGkiOnRydWUsIlYiOiIwLjAuMDAwMCIsIlAiOiJXaW4zMiIsIkFOIjoiTWFpbCIsIldUIjoyfQ%3D%3D%7C0%7C%7C%7C&sdata=AlNlzW4HCLVu3vZ%2BV05rjEpCf32NGo6lfGiKR2MPMLI%3D&reserved=0).

As the corresponding author, your ORCID iD is verified in the submission system and will appear in the published article. PLOS supports the use of ORCID, and we encourage all coauthors to register for an ORCID iD and use it as well. Please encourage your coauthors to verify their ORCID iD within the submission system before final acceptance, as unverified ORCID iDs will not appear in the published article. *Only* the individual author can complete the verification step; PLOS staff *cannot* verify ORCID iDs on behalf of authors.

We look forward to receiving your revised manuscript.

Kind regards,

Juliana Alvarez-Argote
Academic Editor
PLOS One

Journal Requirements:

When submitting your revision, we need you to address these additional requirements.

1.Please ensure that your manuscript meets PLOS ONE's style requirements, including those for file naming. The PLOS ONE style templates can be found at

<https://journals.plos.org/plosone/s/file?id=wjVg/PLOSOne_formatting_sample_main_body.pdf> and

[https://journals.plos.org/plosone/s/file?id=ba62/PLOSOne_formatting_sample_title_authors_affiliations.pdf](https://nam02.safelinks.protection.outlook.com/?url=https%3A%2F%2Ftrack.editorialmanager.com%2FCL0%2Fhttps%3A%252F%252Fjournals.plos.org%252Fplosone%252Fs%252Ffile%253Fid%3Dba62%252FPLOSOne_formatting_sample_title_authors_affiliations.pdf%2F1%2F010f019f3a88c9bf-b8517ac3-d107-4c70-b9d9-95d8bb3339ee-000000%2FiHHQ7Mi7nutw3DYeAhtQMvi5eDjDejPWcG2IpPjRya8%3D258&data=05%7C02%7Cjs5895%40cumc.columbia.edu%7C85eae93336524483ade108dedbd477a5%7Cb0002a9b0017404d97dc3d3bab09be81%7C0%7C0%7C639189903066412857%7CUnknown%7CTWFpbGZsb3d8eyJFbXB0eU1hcGkiOnRydWUsIlYiOiIwLjAuMDAwMCIsIlAiOiJXaW4zMiIsIkFOIjoiTWFpbCIsIldUIjoyfQ%3D%3D%7C0%7C%7C%7C&sdata=DWqCIGXz9wITaVTT2xO9LNNR%2F4eITtmByV8B9spD1gw%3D&reserved=0)

We have updated the format of the manuscript to meet these requirements.

2. Please include a complete copy of PLOS’ questionnaire on inclusivity in global research in your revised manuscript. Our policy for research in this area aims to improve transparency in the reporting of research performed outside of researchers’ own country or community. The policy applies to researchers who have travelled to a different country to conduct research, research with Indigenous populations or their lands, and research on cultural artefacts. The questionnaire can also be requested at the journal’s discretion for any other submissions, even if these conditions are not met.  Please find more information on the policy and a link to download a blank copy of the questionnaire here: <https://journals.plos.org/plosone/s/best-practices-in-research-reporting>. Please upload a completed version of your questionnaire as Supporting Information when you resubmit your manuscript.

We have attached a completed questionnaire as supplementary information.

3. In the online submission form you indicate that your data is not available for proprietary reasons and have provided a contact point for accessing this data. Please note that your current contact point is a co-author on this manuscript. According to our Data Policy, the contact point must not be an author on the manuscript and must be an institutional contact, ideally not an individual. Please revise your data statement to a non-author institutional point of contact, such as a data access or ethics committee, and send this to us via return email. Please also include contact information for the third party organization, and please include the full citation of where the data can be found.

We have now included all the raw data generated in this study as a complete database in S2 Information, which precludes the need to separately deposit data in a repository.

4. If the reviewer comments include a recommendation to cite specific previously published works, please review and evaluate these publications to determine whether they are relevant and should be cited. There is no requirement to cite these works unless the editor has indicated otherwise.

The reviewers did not request any additional citations.

REVIEWER #1:

How was the water intake by dogs documented by dog owners to ensure compliance with the instructions in order to reliably rely on data provided?

Thanks for this question – we provided detailed instructions to dog owners about how to measure water intake over 24 hours by measuring volumes of water in the dog’s bowl at multiple intervals. We also provided a measuring jug with millilitre units to each owner to help with this. However, we did not explicitly check with owners that our instructions were followed correctly. We have added more detailed information about this in the Methods (line 457), as well as adding a limitation in the Discussion (line 299), though we note that measured values and subjective opinions about water intake were significantly correlated in our analysis, which provides at least some internal validation for these measures.

Were the dogs weighted before first water intake?

Dogs were weighed at each follow-up visit, and owners were instructed to perform the water intake measurements within a few days of specified visits. The water intake values were then normalized against the bodyweight measured at the corresponding follow-up visit. We added this information to the Methods (line 457).

What was the standard measure of 6-minute walk by participating dogs?

We walked dogs at a comfortable walking pace on a lead in the same corridor in our hospital, where we had previously measured the length of the corridor. The measure of 6 minute walk was therefore calculated by taking the number of complete “laps” of the corridor, plus any additional distance in a final partial “lap” that was measured with a measuring tape. We have added this information to the methods (line 464).

REVIEWER #2:

line 34, fix "were using".

We corrected this text, thank you.

line 71, consider "promoter(s) such as the GRE (line 279)" instead of "genes".

We have changed to “promoters”.

line 98 or 296, suggest elaborate briefly on the range of dog age, breed, etc.

We have added this information to the text, and a more complete summary is also provided in S1 and S2 Tables.

line 115, I may have missed it but suggest spell out "PCV" on first usage in the text.

We have added this definition at first usage.

line 117 and elsewhere, suggest to avoid subjective terms "greatly, considerably, subtle, marked, dramatically, flagrant", etc.

We have toned town these terms in the text.

line 160 regarding "stimulated" and lines 233/272 "unstimulated", suggest to direct the reader here to Methods line 384 and following.

Thank you for this suggestion, and we provide a link to the Methods.

line 443, I may have missed but I did not find "Supplementary Information 1".

This was provided in first submission but may not have been available to the reviewers. We have noted upon resubmission that this material should be available for review.

REVIEWER #3:

Minor language editing is required to correct occasional typographical and grammatical errors and improve readability in several sections.

Thank you for this feedback. We have revised the manuscript extensively in response to this and other comments, and we are happy to make further adjustments if the reviewer identifies any specific areas for further improvement.

REVIEWER #4:

Major-
1. The data support associations between early changes in selected variables and later outcomes, but the manuscript frequently uses predictive language that seems stronger than warranted for a small observational cohort. I recommend revising claims of “prediction” to emphasize that these findings require validation in a larger independent cohort.

Thank you for this comment, and we agree that it is appropriate to alter the language to focus on associations rather than predictions. We still state that the ability to predict outcomes is a goal of studies like this, but we only describe associations in our results and conclusions. We also updated the title to reflect this change.

2. Of 73 enrolled dogs, 44 were excluded from analysis due to euthanasia or lack of samples. This creates important survivor and selection bias. The limitations should state that the results apply only to dogs that survived to early follow-up with adequate residual samples, and may not apply to the full population of dogs presenting with IMHA.

We agree with this comment, and we expanded the corresponding section in the Discussion to highlight the possible selection bias and need for independent sample validation (line 303).

3. The statistical analysis requires clarification and strengthening. Multiple linear regression with backward AIC-based model selection was performed in a small cohort, with incomplete data for some variables. This approach risks overfitting and unstable predictor selection. Please provide the number of observations included in each final model, full model coefficients, confidence intervals, adjusted R² or other model fit metrics, and diagnostic assessment of model assumptions. If possible, add sensitivity analyses such as bootstrapping, cross-validation, or analyses using simpler prespecified models.

Thank you for this feedback. We agree that any regression models with such small case numbers should be considered exploratory, and we have stated this explicitly throughout the text. Based on the reviewer feedback, we replaced the models with backward selection with simpler prespecified models for both sets of analysis. For time to reach a normal PCV, no variables now show a significant association. For water intake at time T3, ALP and ALT still show the same association, but *Zfp36* expression is not significantly associated. We have updated the results and discussion sections to reflect these changes, and we also provide the additional information suggested by the reviewer for each model in **new S6 and S7 Figures**. On the whole, we feel these models add value to the study by identifying possible candidate associations for follow-up evaluations, but we have greatly toned down the language of the study and taken care to frame these as preliminary results, given the limitations outlined by the reviewer.

4. The statement- "time to reach PCV 35%” needs additional detail. Please clarify how many dogs had interpolated versus extrapolated values? How many values were capped at 90 days? Robustness affected with inclusion vs. exclusion? Also, how were transfustion number/timing handled in analyses?

We have now provided this information in the corresponding results section (line 156). Briefly, values were interpolated in 62% of dogs and extrapolated in 38%, with 10% having assigned values of 90 days. The analysis was not adjusted for transfusions, but we noted that most transfusions were administered in the first 1-2 days after admission to the hospital, and visual inspection of data shown in Fig 3a shows only 1-2 examples where the PCV value increased dramatically at 2^nd^ measurement, then dropped again. We also found no statistical correlation between the inferred time to PCV of 35% and number of transfusions administered. Together, this suggests that early transfusion events have little impact on the predicted time to reach a PCV of 35%. To evaluate the generalizability of this approach, we also identified a further 35 IMHA dogs that had serial PCV measurements, either dogs that were excluded from the main study mainly owing to incomplete residual samples, as well as some dogs presented to the study center after the main study was completed, but which followed the same treatment regimen and follow-up schedule. We applied the same approach in these dogs, finding very similar patterns of change in PCV, as shown in new **S5 Figure**. This independent cohort of dogs had shorter median times to reach a PCV of 35%, though we cannot see any clear reason for this when most dogs would have been included if residual samples would have been available. We note that values in the 2 cohorts show much overlap.

5. Clarify what kind of RT-qPCR machine was used. Only a thermocycler is listed (was qPCR component a Biorad CFX or older MyIQ model?). Please also provide cycling conditions, Cq exclusion criteria, and clearer formatting of the expression calculation.

We have provided this information in the Methods (it was Bio-Rad CFX96) with the cycling conditions and exclusion criteria, along with reformatted equation for expression calculations (line 412 onwards).

6. Reference numbering appears to be incorrect in multiple places. For example, ACVIM diagnostic definition is cited as ref 28, but ref 28 is an azathioprine treatment paper, etc. with multiple other cites. Check numbering for accuracy.

Thank you for pointing this out, and we have updated the reference numbering in this revised manuscript.

7. Please correct all supplementary figure citations and captions. The numbering in manuscript is not lining up with correct sup # in file.

Thank you for pointing this out, and we have updated the numbering in the revised manuscript.

8. Data must be publically available per PLOS guidelines. “Available from the authors upon reasonable request” is not acceptable for access. Please deposit in a public repository.

We now provide a complete data table as **new S2 Information**, which provides all data generated in this study.

9. The direct antiglobulin tests were provided by Alvedia Ltd at reduced cost. This should be included in the author competing interest statement or an explanation given how it does not constitute a competing interest.

This has been added to the author declaration.

10. The manuscript should avoid over-attributing all later clinical changes to prednisolone alone where concurrent disease or medications (i.e. azathioprine, clopidogrel, etc.) could contribute.

Thank you for this important point, and we have adjusted the manuscript to refer more to “treatment-related” AEs rather than specifically to glucocorticoid-related AEs, except where this refers to some specific clinical signs. We also added a specific limitation to this effect in the Discussion (line 307).

Minor-
1. Please define all abbreviations at first use in the main text and figure legends, including AE, HRQoL, NIBP, and RT-qPCR where applicable.

We have now provided definitions for all these abbreviations on first use.

2. Please ensure that the number of dogs included in each figure panel, variable, and regression model is clear, particularly where missing samples, non-detectable analytes, or loss to follow-up reduced the effective sample size below the 29 dogs included in the main analysis.

We would like to note that, in general, all of our graphs show individual data points for each dog, so that the reader can see how many dogs were included in each analysis. We have now additionally added the number of dogs in each column of each graph for extra clarity.

3. Please ensure consistent capitalization of C-reactive protein, packed cell volume, and gene symbols throughout the manuscript and figure legends.

We have now updated these terms in the revised manuscript.

4. Abstract: clincopathological should be clinicopathological.
5. Abstract methods- "with serum cytokine concentrations were using multiplex", maybe "serum cytokine concentrations were measured using multiplex arrays."
6. Line 284- should be "effective control"
7. Line 401- ThermoFisher
8. Line 429- should be intravenously
9. Line 439- Likert scale
10. Figure 3 caption- should be "line represents linear fit" and "P values"

We have corrected and/or updated all the text changes outlined in reviewer points 4-10.
