# Supplementary material for: Dynamic changes in clinicopathological variables and gene expression in dogs with immune-mediated hemolytic anemia
Source: PLoS One. 2026 Oct 28;21(10):e0359710. doi: 10.1371/journal.pone.0359710 (PMC13716216; doi:10.1371/journal.pone.0359710)
Supplement: Supplementary file 6 [file pone.0359710.s016.docx]

Thank you for submitting your manuscript to PLOS One. After careful review, we consider that it has merit but requires some minor revisions to fully meet PLOS One’s publication criteria. Therefore, we invite you to submit a revised version of the manuscript that addresses the points raised during the review process.

Thank you for your assistance in improving the manuscript.

1. Abstract, Conclusions section: Please spell "ALP" [alkaline phosphatase] as it hasn't been spelled in the abstract.

We now define the abbreviation in the abstract.

2. Abstract, Conclusions section: the authors state that "ALP activity could be a useful biomarker of glucocorticoid responsiveness in dogs". However, from the review of the data, the more specific conclusion is that "increasing ALP activity from time T1 to T2 was associated with greater water intake at T3" (line 243) and greater water intake is considered one of the adverse events from glucocorticoid therapy, not efficacy which might be suggested by "glucocorticoid responsiveness". If this is true, can the authors please modify the conclusions to reflect the more precise information? the authors may also want to highlight and incorporate the important remarks that are stated in the conclusion paragraph of the discussion section of the main text (lines 313-316) which seems to me the most valuable point of this manuscript, and which will be important to develop future related studies.

We updated the conclusion to provide this more specific information, and to match the wording of the final part of the discussion.

3. Line 215 and others: Please spell all the abbreviations in the manuscript, such as "ALP and ALT activity" and any other abbreviations. Also it seems that alkaline phosphatase is sometimes used instead of ALP so authors should decide which one they want to state and for consistency only use one form, to avoid confusion to the reader.

We have spelled out ALT and ALP fully, and we believe other abbreviations have now been expanded fully on first use.
