# Supplementary material for: Dynamic changes in clinicopathological variables and gene expression in dogs with immune-mediated hemolytic anemia
Source: PLoS One. 2026 Oct 28;21(10):e0359710. doi: 10.1371/journal.pone.0359710 (PMC13716216; doi:10.1371/journal.pone.0359710)
Supplement: Peer review history

# Peer review: Decision Letter 0

**DOI:** 10.1371/journal.pone.0359710.r001

**Authors:** Juliana Alvarez-Argote

6 Jul 2026

Dear Dr. Swann,

Thank you for submitting your manuscript to PLOS ONE. After careful consideration, we feel that it has merit but does not fully meet PLOS ONE’s publication criteria as it currently stands. Therefore, we invite you to submit a revised version of the manuscript that addresses the points raised during the review process.

Please submit your revised manuscript by Sep 04 2026 11:59PM. If you will need more time than this to complete your revisions, please reply to this message or contact the journal office at plosone@plos.org. When you're ready to submit your revision, log on to https://www.editorialmanager.com/pone/ and select the 'Submissions Needing Revision' folder to locate your manuscript file.

- A letter that responds to each point raised by the academic editor and reviewer(s). You should upload this letter as a separate file labeled 'Response to Reviewers'.
- A marked-up copy of your manuscript that highlights changes made to the original version. You should upload this as a separate file labeled 'Revised Manuscript with Track Changes'.
- An unmarked version of your revised paper without tracked changes. You should upload this as a separate file labeled 'Manuscript'.

If you would like to make changes to your financial disclosure, please include your updated statement in your cover letter. Guidelines for resubmitting your figure files are available below the reviewer comments at the end of this letter.

If applicable, we recommend that you deposit your laboratory protocols in protocols.io to enhance the reproducibility of your results. Protocols.io assigns your protocol its own identifier (DOI) so that it can be cited independently in the future. For instructions see: https://journals.plos.org/plosone/s/submission-guidelines#loc-laboratory-protocols. Additionally, PLOS ONE offers an option for publishing peer-reviewed Lab Protocol articles, which describe protocols hosted on protocols.io. Read more information on sharing protocols at https://plos.org/protocols?utm_medium=editorial-email&utm_source=authorletters&utm_campaign=protocols.

As the corresponding author, your ORCID iD is verified in the submission system and will appear in the published article. PLOS supports the use of ORCID, and we encourage all coauthors to register for an ORCID iD and use it as well. Please encourage your coauthors to verify their ORCID iD within the submission system before final acceptance, as unverified ORCID iDs will not appear in the published article. *Only* the individual author can complete the verification step; PLOS staff *cannot* verify ORCID iDs on behalf of authors.

We look forward to receiving your revised manuscript.

Kind regards,

Juliana Alvarez-Argote

Academic Editor

PLOS One

Journal Requirements:

When submitting your revision, we need you to address these additional requirements.

1.Please ensure that your manuscript meets PLOS ONE's style requirements, including those for file naming. The PLOS ONE style templates can be found at

https://journals.plos.org/plosone/s/file?id=wjVg/PLOSOne_formatting_sample_main_body.pdf and

https://journals.plos.org/plosone/s/file?id=ba62/PLOSOne_formatting_sample_title_authors_affiliations.pdf

2. Please include a complete copy of PLOS’ questionnaire on inclusivity in global research in your revised manuscript. Our policy for research in this area aims to improve transparency in the reporting of research performed outside of researchers’ own country or community. The policy applies to researchers who have travelled to a different country to conduct research, research with Indigenous populations or their lands, and research on cultural artefacts. The questionnaire can also be requested at the journal’s discretion for any other submissions, even if these conditions are not met.  Please find more information on the policy and a link to download a blank copy of the questionnaire here: https://journals.plos.org/plosone/s/best-practices-in-research-reporting. Please upload a completed version of your questionnaire as Supporting Information when you resubmit your manuscript.

3. In the online submission form you indicate that your data is not available for proprietary reasons and have provided a contact point for accessing this data. Please note that your current contact point is a co-author on this manuscript. According to our Data Policy, the contact point must not be an author on the manuscript and must be an institutional contact, ideally not an individual. Please revise your data statement to a non-author institutional point of contact, such as a data access or ethics committee, and send this to us via return email. Please also include contact information for the third party organization, and please include the full citation of where the data can be found.

4. If the reviewer comments include a recommendation to cite specific previously published works, please review and evaluate these publications to determine whether they are relevant and should be cited. There is no requirement to cite these works unless the editor has indicated otherwise.

Additional Editor Comments:

How was the water intake by dogs documented by dog owners to ensure compliance with the instructions in order to reliably rely on data provided?

Were the dogs weighted before first water intake? What was the standard measure of 6-minute walk by participating dogs?

line 34, fix "were using".

line 71, consider "promoter(s) such as the GRE (line 279)" instead of "genes".

line 98 or 296, suggest elaborate briefly on the range of dog age, breed, etc.

line 115, I may have missed it but suggest spell out "PCV" on first usage in the text.

line 117 and elsewhere, suggest to avoid subjective terms "greatly, considerably, subtle, marked, dramatically, flagrant", etc.

line 160 regarding "stimulated" and lines 233/272 "unstimulated", suggest to direct the reader here to Methods line 384 and following.

REVIEWER #4:

Major-

1. The data support associations between early changes in selected variables and later outcomes, but the manuscript frequently uses predictive language that seems stronger than warranted for a small observational cohort. I recommend revising claims of “prediction” to emphasize that these findings require validation in a larger independent cohort.

2. Of 73 enrolled dogs, 44 were excluded from analysis due to euthanasia or lack of samples. This creates important survivor and selection bias. The limitations should state that the results apply only to dogs that survived to early follow-up with adequate residual samples, and may not apply to the full population of dogs presenting with IMHA.

3. The statistical analysis requires clarification and strengthening. Multiple linear regression with backward AIC-based model selection was performed in a small cohort, with incomplete data for some variables. This approach risks overfitting and unstable predictor selection. Please provide the number of observations included in each final model, full model coefficients, confidence intervals, adjusted R² or other model fit metrics, and diagnostic assessment of model assumptions. If possible, add sensitivity analyses such as bootstrapping, cross-validation, or analyses using simpler prespecified models.

4. The statement- "time to reach PCV 35%” needs additional detail. Please clarify how many dogs had interpolated versus extrapolated values? How many values were capped at 90 days? Robustness affected with inclusion vs. exclusion? Also, how were transfustion number/timing handled in analyses?

5. Clarify what kind of RT-qPCR machine was used. Only a thermocycler is listed (was qPCR component a Biorad CFX or older MyIQ model?). Please also provide cycling conditions, Cq exclusion criteria, and clearer formatting of the expression calculation.

6. Reference numbering appears to be incorrect in multiple places. For example, ACVIM diagnostic definition is cited as ref 28, but ref 28 is an azathioprine treatment paper, etc. with multiple other cites. Check numbering for accuracy.

7. Please correct all supplementary figure citations and captions. The numbering in manuscript is not lining up with correct sup # in file.

8. Data must be publically available per PLOS guidelines. “Available from the authors upon reasonable request” is not acceptable for access. Please deposit in a public repository.

9. The direct antiglobulin tests were provided by Alvedia Ltd at reduced cost. This should be included in the author competing interest statement or an explanation given how it does not constitute a competing interest.

10. The manuscript should avoid over-attributing all later clinical changes to prednisolone alone where concurrent disease or medications (i.e. azathioprine, clopidogrel, etc.) could contribute.

Minor-

1. Please define all abbreviations at first use in the main text and figure legends, including AE, HRQoL, NIBP, and RT-qPCR where applicable.

2. Please ensure that the number of dogs included in each figure panel, variable, and regression model is clear, particularly where missing samples, non-detectable analytes, or loss to follow-up reduced the effective sample size below the 29 dogs included in the main analysis.

3. Please ensure consistent capitalization of C-reactive protein, packed cell volume, and gene symbols throughout the manuscript and figure legends.

4. Abstract: clincopathological should be clinicopathological.

5. Abstract methods- "with serum cytokine concentrations were using multiplex", maybe "serum cytokine concentrations were measured using multiplex arrays."

6. Line 284- should be "effective control"

7. Line 401- ThermoFisher

8. Line 429- should be intravenously

9. Line 439- Likert scale

[Note: HTML markup is below. Please do not edit.]

Reviewers' comments:

Reviewer's Responses to Questions

**Comments to the Author**

1. Is the manuscript technically sound, and do the data support the conclusions?

Reviewer #1: Yes

Reviewer #2: Yes

Reviewer #3: Yes

Reviewer #4: Yes

**********

2. Has the statistical analysis been performed appropriately and rigorously?

Reviewer #1: Yes

Reviewer #2: Yes

Reviewer #3: Yes

Reviewer #4: No

**********

3. Have the authors made all data underlying the findings in their manuscript fully available?

The PLOS Data policy

Reviewer #1: Yes

Reviewer #2: Yes

Reviewer #3: Yes

Reviewer #4: No

**********

4. Is the manuscript presented in an intelligible fashion and written in standard English?

Reviewer #1: Yes

Reviewer #2: Yes

Reviewer #3: Yes

Reviewer #4: Yes

**********

Reviewer #1: How was the water intake by dogs documented by dog owners to ensure compliance with the instructions in order to reliably rely on data provided?

Were the dogs weighted before first water intake? What was the standard measure of 6-minute walk by participating dogs?

Reviewer #2: This well-written manuscript describes observational quantitative studies of a canine cohort with clinical IMHA and responses to standard treatment as accurately reflected in the title. Authors conclude that certain clinical observations and correlated gene expression levels may be useful to predict the course and outcomes of this disease, while other variables measured will likely not.

The work was done by investigators who are well-established in this specialty and were well-positioned to conduct the work.

Minor edits suggested:

line 34, fix "were using".

line 71, consider "promoter(s) such as the GRE (line 279)" instead of "genes".

line 98 or 296, suggest elaborate briefly on the range of dog age, breed, etc.

line 115, I may have missed it but suggest spell out "PCV" on first usage in the text.

line 117 and elsewhere, suggest to avoid subjective terms "greatly, considerably, subtle, marked, dramatically, flagrant", etc.

line 160 regarding "stimulated" and lines 233/272 "unstimulated", suggest to direct the reader here to Methods line 384 and following.

line 443, I may have missed but I did not find "Supplementary Information 1".

Reviewer #3: The manuscript presents a prospective observational study investigating dynamic changes in clinicopathological variables and gene expression during treatment of canine immune-mediated hemolytic anemia (IMHA). The topic is clinically relevant, and the authors address an important unmet need in veterinary medicine: identification of biomarkers that may predict recovery from anemia and the development of glucocorticoid-related adverse effects. The study is generally well designed, and the manuscript is clearly structured.

The diagnostic criteria, inclusion and exclusion criteria, laboratory methods, and statistical approaches are described in sufficient detail. The longitudinal study design and repeated measurements provide valuable information regarding temporal changes during treatment. The identification of serum bilirubin, alkaline phosphatase activity, and Zfp36 expression as potential predictors of treatment response and adverse effects is interesting and may warrant further investigation.

The manuscript is generally written in clear and understandable English. Only minor language editing is required to correct occasional typographical and grammatical errors and improve readability in several sections.

Overall, this is a technically sound and clinically relevant study. The conclusions are generally supported by the data presented, although they should be interpreted as preliminary until validated in larger independent cohorts. I believe the manuscript would be suitable for publication.

Reviewer #4: This prospective observational study evaluates clinicopathologic, immune, and glucocorticoid-regulated gene expression changes in dogs with non-associative immune-mediated hemolytic anemia. The work addresses an important clinical problem, namely the need to individualize treatment in dogs with IMHA by identifying early markers of hematologic recovery and later glucocorticoid-associated adverse effects. If revised, the study could provide useful data and may guide future studies of biomarker-driven treatment adjustment in canine IMHA.

Major-

1. The data support associations between early changes in selected variables and later outcomes, but the manuscript frequently uses predictive language that seems stronger than warranted for a small observational cohort. I recommend revising claims of “prediction” to emphasize that these findings require validation in a larger independent cohort.

2. Of 73 enrolled dogs, 44 were excluded from analysis due to euthanasia or lack of samples. This creates important survivor and selection bias. The limitations should state that the results apply only to dogs that survived to early follow-up with adequate residual samples, and may not apply to the full population of dogs presenting with IMHA.

3. The statistical analysis requires clarification and strengthening. Multiple linear regression with backward AIC-based model selection was performed in a small cohort, with incomplete data for some variables. This approach risks overfitting and unstable predictor selection. Please provide the number of observations included in each final model, full model coefficients, confidence intervals, adjusted R² or other model fit metrics, and diagnostic assessment of model assumptions. If possible, add sensitivity analyses such as bootstrapping, cross-validation, or analyses using simpler prespecified models.

4. The statement- "time to reach PCV 35%” needs additional detail. Please clarify how many dogs had interpolated versus extrapolated values? How many values were capped at 90 days? Robustness affected with inclusion vs. exclusion? Also, how were transfustion number/timing handled in analyses?

5. Clarify what kind of RT-qPCR machine was used. Only a thermocycler is listed (was qPCR component a Biorad CFX or older MyIQ model?). Please also provide cycling conditions, Cq exclusion criteria, and clearer formatting of the expression calculation.

6. Reference numbering appears to be incorrect in multiple places. For example, ACVIM diagnostic definition is cited as ref 28, but ref 28 is an azathioprine treatment paper, etc. with multiple other cites. Check numbering for accuracy.

7. Please correct all supplementary figure citations and captions. The numbering in manuscript is not lining up with correct sup # in file.

8. Data must be publically available per PLOS guidelines. “Available from the authors upon reasonable request” is not acceptable for access. Please deposit in a public repository.

9. The direct antiglobulin tests were provided by Alvedia Ltd at reduced cost. This should be included in the author competing interest statement or an explanation given how it does not constitute a competing interest.

10. The manuscript should avoid over-attributing all later clinical changes to prednisolone alone where concurrent disease or medications (i.e. azathioprine, clopidogrel, etc.) could contribute.

Minor-

1. Please define all abbreviations at first use in the main text and figure legends, including AE, HRQoL, NIBP, and RT-qPCR where applicable.

2. Please ensure that the number of dogs included in each figure panel, variable, and regression model is clear, particularly where missing samples, non-detectable analytes, or loss to follow-up reduced the effective sample size below the 29 dogs included in the main analysis.

3. Please ensure consistent capitalization of C-reactive protein, packed cell volume, and gene symbols throughout the manuscript and figure legends.

4. Abstract: clincopathological should be clinicopathological.

5. Abstract methods- "with serum cytokine concentrations were using multiplex", maybe "serum cytokine concentrations were measured using multiplex arrays."

6. Line 284- should be "effective control"

7. Line 401- ThermoFisher

8. Line 429- should be intravenously

9. Line 439- Likert scale

10. Figure 3 caption- should be "line represents linear fit" and "P values"

**********

what does this mean?). If published, this will include your full peer review and any attached files.

If you choose “no”, your identity will remain anonymous but your review may still be made public.

**Do you want your identity to be public for this peer review?** For information about this choice, including consent withdrawal, please see our Privacy Policy

Reviewer #1: **Yes:**  PRINCE ADDO ANIM

Reviewer #2: No

Reviewer #3: No

Reviewer #4: No

**********

[NOTE: If reviewer comments were submitted as an attachment file, they will be attached to this email and accessible via the submission site. Please log into your account, locate the manuscript record, and check for the action link "View Attachments". If this link does not appear, there are no attachment files.]

To ensure your figures meet our technical requirements, please review our figure guidelines: https://journals.plos.org/plosone/s/figures

You may also use PLOS’s free figure tool, NAAS, to help you prepare publication quality figures: https://journals.plos.org/plosone/s/figures#loc-tools-for-figure-preparation.

NAAS will assess whether your figures meet our technical requirements by comparing each figure against our figure specifications.

# Author comment: Author response to Decision Letter 1

**DOI:** 10.1371/journal.pone.0359710.r002

27 Aug 2026

PONE-D-26-22460

Dynamic changes in clinicopathological variables and gene expression predict recovery from anemia and treatment-related adverse effects in dogs with immune-mediated hemolytic anemia

PLOS One

Dear Dr. Swann,

Thank you for submitting your manuscript to PLOS ONE. After careful consideration, we feel that it has merit but does not fully meet PLOS ONE’s publication criteria as it currently stands. Therefore, we invite you to submit a revised version of the manuscript that addresses the points raised during the review process.

Please submit your revised manuscript by Sep 04 2026 11:59PM. If you will need more time than this to complete your revisions, please reply to this message or contact the journal office at plosone@plos.org. When you're ready to submit your revision, log on to https://www.editorialmanager.com/pone/ and select the 'Submissions Needing Revision' folder to locate your manuscript file.

Please include the following items when submitting your revised manuscript:

• A letter that responds to each point raised by the academic editor and reviewer(s). You should upload this letter as a separate file labeled 'Response to Reviewers'.

• A marked-up copy of your manuscript that highlights changes made to the original version. You should upload this as a separate file labeled 'Revised Manuscript with Track Changes'.

• An unmarked version of your revised paper without tracked changes. You should upload this as a separate file labeled 'Manuscript'.

If you would like to make changes to your financial disclosure, please include your updated statement in your cover letter. Guidelines for resubmitting your figure files are available below the reviewer comments at the end of this letter.

If applicable, we recommend that you deposit your laboratory protocols in protocols.io to enhance the reproducibility of your results. Protocols.io assigns your protocol its own identifier (DOI) so that it can be cited independently in the future. For instructions see: https://journals.plos.org/plosone/s/submission-guidelines#loc-laboratory-protocols. Additionally, PLOS ONE offers an option for publishing peer-reviewed Lab Protocol articles, which describe protocols hosted on protocols.io. Read more information on sharing protocols at https://plos.org/protocols?utm_medium=editorial-email&utm_source=authorletters&utm_campaign=protocols.

As the corresponding author, your ORCID iD is verified in the submission system and will appear in the published article. PLOS supports the use of ORCID, and we encourage all coauthors to register for an ORCID iD and use it as well. Please encourage your coauthors to verify their ORCID iD within the submission system before final acceptance, as unverified ORCID iDs will not appear in the published article. Only the individual author can complete the verification step; PLOS staff cannot verify ORCID iDs on behalf of authors.

We look forward to receiving your revised manuscript.

Kind regards,

Juliana Alvarez-Argote

Academic Editor

PLOS One

Journal Requirements:

When submitting your revision, we need you to address these additional requirements.

1.Please ensure that your manuscript meets PLOS ONE's style requirements, including those for file naming. The PLOS ONE style templates can be found at

https://journals.plos.org/plosone/s/file?id=wjVg/PLOSOne_formatting_sample_main_body.pdf and

https://journals.plos.org/plosone/s/file?id=ba62/PLOSOne_formatting_sample_title_authors_affiliations.pdf

We have updated the format of the manuscript to meet these requirements.

2. Please include a complete copy of PLOS’ questionnaire on inclusivity in global research in your revised manuscript. Our policy for research in this area aims to improve transparency in the reporting of research performed outside of researchers’ own country or community. The policy applies to researchers who have travelled to a different country to conduct research, research with Indigenous populations or their lands, and research on cultural artefacts. The questionnaire can also be requested at the journal’s discretion for any other submissions, even if these conditions are not met. Please find more information on the policy and a link to download a blank copy of the questionnaire here: https://journals.plos.org/plosone/s/best-practices-in-research-reporting. Please upload a completed version of your questionnaire as Supporting Information when you resubmit your manuscript.

We have attached a completed questionnaire as supplementary information.

3. In the online submission form you indicate that your data is not available for proprietary reasons and have provided a contact point for accessing this data. Please note that your current contact point is a co-author on this manuscript. According to our Data Policy, the contact point must not be an author on the manuscript and must be an institutional contact, ideally not an individual. Please revise your data statement to a non-author institutional point of contact, such as a data access or ethics committee, and send this to us via return email. Please also include contact information for the third party organization, and please include the full citation of where the data can be found.

We have now included all the raw data generated in this study as a complete database in S2 Information, which precludes the need to separately deposit data in a repository.

4. If the reviewer comments include a recommendation to cite specific previously published works, please review and evaluate these publications to determine whether they are relevant and should be cited. There is no requirement to cite these works unless the editor has indicated otherwise.

The reviewers did not request any additional citations.

REVIEWER #1:

How was the water intake by dogs documented by dog owners to ensure compliance with the instructions in order to reliably rely on data provided?

Thanks for this question – we provided detailed instructions to dog owners about how to measure water intake over 24 hours by measuring volumes of water in the dog’s bowl at multiple intervals. We also provided a measuring jug with millilitre units to each owner to help with this. However, we did not explicitly check with owners that our instructions were followed correctly. We have added more detailed information about this in the Methods (line 457), as well as adding a limitation in the Discussion (line 299), though we note that measured values and subjective opinions about water intake were significantly correlated in our analysis, which provides at least some internal validation for these measures.

Were the dogs weighted before first water intake?

Dogs were weighed at each follow-up visit, and owners were instructed to perform the water intake measurements within a few days of specified visits. The water intake values were then normalized against the bodyweight measured at the corresponding follow-up visit. We added this information to the Methods (line 457).

What was the standard measure of 6-minute walk by participating dogs?

We walked dogs at a comfortable walking pace on a lead in the same corridor in our hospital, where we had previously measured the length of the corridor. The measure of 6 minute walk was therefore calculated by taking the number of complete “laps” of the corridor, plus any additional distance in a final partial “lap” that was measured with a measuring tape. We have added this information to the methods (line 464).

REVIEWER #2:

line 34, fix "were using".

We corrected this text, thank you.

line 71, consider "promoter(s) such as the GRE (line 279)" instead of "genes".

We have changed to “promoters”.

line 98 or 296, suggest elaborate briefly on the range of dog age, breed, etc.

We have added this information to the text, and a more complete summary is also provided in S1 and S2 Tables.

line 115, I may have missed it but suggest spell out "PCV" on first usage in the text.

We have added this definition at first usage.

line 117 and elsewhere, suggest to avoid subjective terms "greatly, considerably, subtle, marked, dramatically, flagrant", etc.

We have toned town these terms in the text.

line 160 regarding "stimulated" and lines 233/272 "unstimulated", suggest to direct the reader here to Methods line 384 and following.

Thank you for this suggestion, and we provide a link to the Methods.

line 443, I may have missed but I did not find "Supplementary Information 1".

This was provided in first submission but may not have been available to the reviewers. We have noted upon resubmission that this material should be available for review.

REVIEWER #3:

Minor language editing is required to correct occasional typographical and grammatical errors and improve readability in several sections.

Thank you for this feedback. We have revised the manuscript extensively in response to this and other comments, and we are happy to make further adjustments if the reviewer identifies any specific areas for further improvement.

REVIEWER #4:

Major-

1. The data support associations between early changes in selected variables and later outcomes, but the manuscript frequently uses predictive language that seems stronger than warranted for a small observational cohort. I recommend revising claims of “prediction” to emphasize that these findings require validation in a larger independent cohort.

Thank you for this comment, and we agree that it is appropriate to alter the language to focus on associations rather than predictions. We still state that the ability to predict outcomes is a goal of studies like this, but we only describe associations in our results and conclusions. We also updated the title to reflect this change.

2. Of 73 enrolled dogs, 44 were excluded from analysis due to euthanasia or lack of samples. This creates important survivor and selection bias. The limitations should state that the results apply only to dogs that survived to early follow-up with adequate residual samples, and may not apply to the full population of dogs presenting with IMHA.

We agree with this comment, and we expanded the corresponding section in the Discussion to highlight the possible selection bias and need for independent sample validation (line 303).

3. The statistical analysis requires clarification and strengthening. Multiple linear regression with backward AIC-based model selection was performed in a small cohort, with incomplete data for some variables. This approach risks overfitting and unstable predictor selection. Please provide the number of observations included in each final model, full model coefficients, confidence intervals, adjusted R² or other model fit metrics, and diagnostic assessment of model assumptions. If possible, add sensitivity analyses such as bootstrapping, cross-validation, or analyses using simpler prespecified models.

Thank you for this feedback. We agree that any regression models with such small case numbers should be considered exploratory, and we have stated this explicitly throughout the text. Based on the reviewer feedback, we replaced the models with backward selection with simpler prespecified models for both sets of analysis. For time to reach a normal PCV, no variables now show a significant association. For water intake at time T3, ALP and ALT still show the same association, but Zfp36 expression is not significantly associated. We have updated the results and discussion sections to reflect these changes, and we also provide the additional information suggested by the reviewer for each model in new S6 and S7 Figures. On the whole, we feel these models add value to the study by identifying possible candidate associations for follow-up evaluations, but we have greatly toned down the language of the study and taken care to frame these as preliminary results, given the limitations outlined by the reviewer.

4. The statement- "time to reach PCV 35%” needs additional detail. Please clarify how many dogs had interpolated versus extrapolated values? How many values were capped at 90 days? Robustness affected with inclusion vs. exclusion? Also, how were transfustion number/timing handled in analyses?

We have now provided this information in the corresponding results section (line 156). Briefly, values were interpolated in 62% of dogs and extrapolated in 38%, with 10% having assigned values of 90 days. The analysis was not adjusted for transfusions, but we noted that most transfusions were administered in the first 1-2 days after admission to the hospital, and visual inspection of data shown in Fig 3a shows only 1-2 examples where the PCV value increased dramatically at 2nd measurement, then dropped again. We also found no statistical correlation between the inferred time to PCV of 35% and number of transfusions administered. Together, this suggests that early transfusion events have little impact on the predicted time to reach a PCV of 35%. To evaluate the generalizability of this approach, we also identified a further 35 IMHA dogs that had serial PCV measurements, either dogs that were excluded from the main study mainly owing to incomplete residual samples, as well as some dogs presented to the study center after the main study was completed, but which followed the same treatment regimen and follow-up schedule. We applied the same approach in these dogs, finding very similar patterns of change in PCV, as shown in new S5 Figure. This independent cohort of dogs had shorter median times to reach a PCV of 35%, though we cannot see any clear reason for this when most dogs would have been included if residual samples would have been available. We note that values in the 2 cohorts show much overlap.

5. Clarify what kind of RT-qPCR machine was used. Only a thermocycler is listed (was qPCR component a Biorad CFX or older MyIQ model?). Please also provide cycling conditions, Cq exclusion criteria, and clearer formatting of the expression calculation.

We have provided this information in the Methods (it was Bio-Rad CFX96) with the cycling conditions and exclusion criteria, along with reformatted equation for expression calculations (line 412 onwards).

6. Reference numbering appears to be incorrect in multiple places. For example, ACVIM diagnostic definition is cited as ref 28, but ref 28 is an azathioprine treatment paper, etc. with multiple other cites. Check numbering for accuracy.

Thank you for pointing this out, and we have updated the reference numbering in this revised manuscript.

7. Please correct all supplementary figure citations and captions. The numbering in manuscript is not lining up with correct sup # in file.

Thank you for pointing this out, and we have updated the numbering in the revised manuscript.

8. Data must be publically available per PLOS guidelines. “Available from the authors upon reasonable request” is not acceptable for access. Please deposit in a public repository.

We now provide a complete data table as new S2 Information, which provides all data generated in this study.

9. The direct antiglobulin tests were provided by Alvedia Ltd at reduced cost. This should be included in the author competing interest statement or an explanation given how it does not constitute a competing interest.

This has been added to the author declaration.

10. The manuscript should avoid over-attributing all later clinical changes to prednisolone alone where concurrent disease or medications (i.e. azathioprine, clopidogrel, etc.) could contribute.

Thank you for this important point, and we have adjusted the manuscript to refer more to “treatment-related” AEs rather than specifically to glucocorticoid-related AEs, except where this refers to some specific clinical signs. We also added a specific limitation to this effect in the Discussion (line 307).

Minor-

1. Please define all abbreviations at first use in the main text and figure legends, including AE, HRQoL, NIBP, and RT-qPCR where applicable.

We have now provided definitions for all these abbreviations on first use.

2. Please ensure that the number of dogs included in each figure panel, variable, and regression model is clear, particularly where missing samples, non-detectable analytes, or loss to follow-up reduced the effective sample size below the 29 dogs included in the main analysis.

We would like to note that, in general, all of our graphs show individual data points for each dog, so that the reader can see how many dogs were included in each analysis. We have now additionally added the number of dogs in each column of each graph for extra clarity.

3. Please ensure consistent capitalization of C-reactive protein, packed cell volume, and gene symbols throughout the manuscript and figure legends.

We have now updated these terms in the revised manuscript.

4. Abstract: clincopathological should be clinicopathological.

5. Abstract methods- "with serum cytokine concentrations were using multiplex", maybe "serum cytokine concentrations were measured using multiplex arrays."

6. Line 284- should be "effective control"

7. Line 401- ThermoFisher

8. Line 429- should be intravenously

9. Line 439- Likert scale

10. Figure 3 caption- should be "line represents linear fit" and "P values"

We have corrected and/or updated all the text changes outlined in reviewer points 4-10.

Attachment Submitted filename: 260719 Response to reviewer comments.docx

# Editor report: Decision Letter 1

**DOI:** 10.1371/journal.pone.0359710.r003

**Authors:** Juliana Alvarez-Argote

3 Sep 2026

Dear Dr. Swann,

1. Abstract, Conclusions section: the authors state that "ALP activity could be a useful biomarker of glucocorticoid responsiveness in dogs". However, from the review of the data, the more specific conclusion is that "increasing ALP activity from time T1 to T2 was associated with greater water intake at T3" (line 243) and greater water intake is considered one of the adverse events from glucocorticoid therapy, not efficacy which might be suggested by "glucocorticoid responsiveness". If this is true, can the authors please modify the conclusions to reflect the more precise information? the authors may also want to highlight and incorporate the important remarks that are stated in the conclusion paragraph of the discussion section of the main text (lines 313-316) which seems to me the most valuable point of this manuscript, and which will be important to develop future related studies.
2. Line 215 and others: Please spell all the abbreviations in the manuscript, such as "ALP and ALT activity" and any other abbreviations. Also it seems that alkaline phosphatase is sometimes used instead of ALP so authors should decide which one they want to state and for consistency only use one form, to avoid confusion to the reader.

plosone@plos.org. When you're ready to submit your revision, log on to https://www.editorialmanager.com/pone/ and select the 'Submissions Needing Revision' folder to locate your manuscript file.

- A letter that responds to each point raised by the academic editor and reviewer(s). You should upload this letter as a separate file labeled 'Response to Reviewers'.
- A marked-up copy of your manuscript that highlights changes made to the original version. You should upload this as a separate file labeled 'Revised Manuscript with Track Changes'.
- An unmarked version of your revised paper without tracked changes. You should upload this as a separate file labeled 'Manuscript'.

If you would like to make changes to your financial disclosure, please include your updated statement in your cover letter. Guidelines for resubmitting your figure files are available below the reviewer comments at the end of this letter.

If applicable, we recommend that you deposit your laboratory protocols in protocols.io to enhance the reproducibility of your results. Protocols.io assigns your protocol its own identifier (DOI) so that it can be cited independently in the future. For instructions see: https://journals.plos.org/plosone/s/submission-guidelines#loc-laboratory-protocols. Additionally, PLOS One offers an option for publishing peer-reviewed Lab Protocol articles, which describe protocols hosted on protocols.io. Read more information on sharing protocols at https://plos.org/protocols?utm_medium=editorial-email&utm_source=authorletters&utm_campaign=protocols.

As the corresponding author, your ORCID iD is verified in the submission system and will appear in the published article. PLOS supports the use of ORCID, and we encourage all coauthors to register for an ORCID iD and use it as well. Please encourage your coauthors to verify their ORCID iD within the submission system before final acceptance, as unverified ORCID iDs will not appear in the published article. *Only* the individual author can complete the verification step; PLOS staff *cannot* verify ORCID iDs on behalf of authors.

We look forward to receiving your revised manuscript.

Kind regards,

Juliana Alvarez-Argote

Academic Editor

PLOS One

Journal Requirements:

Please review your reference list to ensure that it is complete and correct. If you have cited papers that have been retracted, please include the rationale for doing so in the manuscript text, or remove these references and replace them with relevant current references. Any changes to the reference list should be mentioned in the rebuttal letter that accompanies your revised manuscript. If you need to cite a retracted article, indicate the article’s retracted status in the References list and also include a citation and full reference for the retraction notice.

**Additional Editor Comments:**

Please address the three minor revision points above.

[NOTE: If reviewer comments were submitted as an attachment file, they will be attached to this email and accessible via the submission site. Please log into your account, locate the manuscript record, and check for the action link "View Attachments". If this link does not appear, there are no attachment files.]

To ensure your figures meet our technical requirements, please review our figure guidelines: https://journals.plos.org/plosone/s/figures

You may also use PLOS’s free figure tool, NAAS, to help you prepare publication quality figures: https://journals.plos.org/plosone/s/figures#loc-tools-for-figure-preparation.

NAAS will assess whether your figures meet our technical requirements by comparing each figure against our figure specifications.

# Author comment: Author response to Decision Letter 2

**DOI:** 10.1371/journal.pone.0359710.r004

4 Sep 2026

Thank you for submitting your manuscript to PLOS One. After careful review, we consider that it has merit but requires some minor revisions to fully meet PLOS One’s publication criteria. Therefore, we invite you to submit a revised version of the manuscript that addresses the points raised during the review process.

Thank you for your assistance in improving the manuscript.

1. Abstract, Conclusions section: Please spell "ALP" [alkaline phosphatase] as it hasn't been spelled in the abstract.

We now define the abbreviation in the abstract.

2. Abstract, Conclusions section: the authors state that "ALP activity could be a useful biomarker of glucocorticoid responsiveness in dogs". However, from the review of the data, the more specific conclusion is that "increasing ALP activity from time T1 to T2 was associated with greater water intake at T3" (line 243) and greater water intake is considered one of the adverse events from glucocorticoid therapy, not efficacy which might be suggested by "glucocorticoid responsiveness". If this is true, can the authors please modify the conclusions to reflect the more precise information? the authors may also want to highlight and incorporate the important remarks that are stated in the conclusion paragraph of the discussion section of the main text (lines 313-316) which seems to me the most valuable point of this manuscript, and which will be important to develop future related studies.

We updated the conclusion to provide this more specific information, and to match the wording of the final part of the discussion.

3. Line 215 and others: Please spell all the abbreviations in the manuscript, such as "ALP and ALT activity" and any other abbreviations. Also it seems that alkaline phosphatase is sometimes used instead of ALP so authors should decide which one they want to state and for consistency only use one form, to avoid confusion to the reader.

We have spelled out ALT and ALP fully, and we believe other abbreviations have now been expanded fully on first use.

Attachment Submitted filename: 260904 Response to reviewer comments.docx

# Editor report: Decision Letter 2

**DOI:** 10.1371/journal.pone.0359710.r005

**Authors:** Juliana Alvarez-Argote

17 Sep 2026

Dynamic changes in clinicopathological variables and gene expression in dogs with immune-mediated hemolytic anemia

PONE-D-26-22460R2

Dear Dr. Swann,

We’re pleased to inform you that your manuscript has been judged scientifically suitable for publication and will be formally accepted for publication once it meets all outstanding technical requirements.

Within one week, you’ll receive an e-mail detailing the required amendments. When these have been addressed, you’ll receive a formal acceptance letter and your manuscript will be scheduled for publication.

An invoice will be generated when your article is formally accepted. Please note, if your institution has a publishing partnership with PLOS and your article meets the relevant criteria, all or part of your publication costs will be covered. Please make sure your user information is up-to-date by logging into Editorial Manager at Editorial Manager® and clicking the ‘Update My Information' link at the top of the page. For questions related to billing, please contact billing support.

If your institution or institutions have a press office, please notify them about your upcoming paper to help maximize its impact. If they’ll be preparing press materials, please inform our press team as soon as possible -- no later than 48 hours after receiving the formal acceptance. Your manuscript will remain under strict press embargo until 2 pm Eastern Time on the date of publication. For more information, please contact onepress@plos.org.

Kind regards,

Juliana Alvarez-Argote

Academic Editor

PLOS One

# Editor report: Acceptance letter

**DOI:** 10.1371/journal.pone.0359710.r006

**Authors:** Juliana Alvarez-Argote

PONE-D-26-22460R2

PLOS One

Dear Dr. Swann,

I'm pleased to inform you that your manuscript has been deemed suitable for publication in PLOS One. Congratulations! Your manuscript is now being handed over to our production team.

At this stage, our production department will prepare your paper for publication. This includes ensuring the following:

* All references, tables, and figures are properly cited

* All relevant supporting information is included in the manuscript submission,

* There are no issues that prevent the paper from being properly typeset

You will receive further instructions from the production team, including instructions on how to review your proof when it is ready. Please keep in mind that we are working through a large volume of accepted articles, so please give us a few days to review your paper and let you know the next and final steps.

Lastly, if your institution or institutions have a press office, please let them know about your upcoming paper now to help maximize its impact. If they'll be preparing press materials, please inform our press team within the next 48 hours. Your manuscript will remain under strict press embargo until 2 pm Eastern Time on the date of publication. For more information, please contact onepress@plos.org.

You will receive an invoice from PLOS for your publication fee after your manuscript has reached the completed accept phase. If you receive an email requesting payment before acceptance or for any other service, this may be a phishing scheme. Learn how to identify phishing emails and protect your accounts at https://explore.plos.org/phishing.

If we can help with anything else, please email us at customercare@plos.org.

Thank you for submitting your work to PLOS One and supporting open access.

Kind regards,

PLOS One Editorial Office Staff

on behalf of

Dr. Juliana Alvarez-Argote

Academic Editor

PLOS One
